# Shelf-Life Stability of Ready-to-Use Green Rooibos Iced Tea Powder—Assessment of Physical, Chemical, and Sensory Properties

**DOI:** 10.3390/molecules26175260

**Published:** 2021-08-30

**Authors:** Chantelle Human, Dalene de Beer, Magdalena Muller, Marieta van der Rijst, Marique Aucamp, Andreas Tredoux, André de Villiers, Elizabeth Joubert

**Affiliations:** 1 Plant Bioactives Group, Post-Harvest and Agro-Processing Technologies, Agricultural Research Council (Infruitec-Nietvoorbij), Private Bag X5026, Stellenbosch 7599, South Africa; DBeerD@arc.agric.za (D.d.B.); JoubertL@arc.agric.za (E.J.); 2 Department of Food Science, Stellenbosch University, Private Bag X1, Stellenbosch 7600, South Africa; mm7@sun.ac.za; 3 Biometry Unit, Agricultural Research Council, Private Bag X5026, Stellenbosch 7599, South Africa; VanDerRijstM@arc.agric.za; 4 School of Pharmacy, University of the Western Cape, Private Bag X17, Bellville 7535, South Africa; maucamp@uwc.ac.za; 5 Department of Chemistry and Polymer Science, Stellenbosch University, Private Bag X1, Stellenbosch 7600, South Africa; atredoux@sun.ac.za (A.T.); ajdevill@sun.ac.za (A.d.V.)

**Keywords:** rooibos iced tea, beverage powder, storage stability, aspalathin, sensory profile, volatile compounds

## Abstract

Green rooibos extract (GRE), shown to improve hyperglycemia and HDL/LDL blood cholesterol, has potential as a nutraceutical beverage ingredient. The main bioactive compound of the extract is aspalathin, a *C*-glucosyl dihydrochalcone. The study aimed to determine the effect of common iced tea ingredients (citric acid, ascorbic acid, and xylitol) on the stability of GRE, microencapsulated with inulin for production of a powdered beverage. The stability of the powder mixtures stored in semi-permeable (5 months) and impermeable (12 months) single-serve packaging at 30 °C and 40 °C/65% relative humidity was assessed. More pronounced clumping and darkening of the powders, in combination with higher first order reaction rate constants for dihydrochalcone degradation, indicated the negative effect of higher storage temperature and an increase in moisture content when stored in the semi-permeable packaging. These changes were further increased by the addition of crystalline ingredients, especially citric acid monohydrate. The sensory profile of the powders (reconstituted to beverage strength iced tea solutions) changed with storage from a predominant green-vegetal aroma to a fruity-sweet aroma, especially when stored at 40 °C/65% RH in the semi-permeable packaging. The change in the sensory profile of the powder mixtures could be attributed to a decrease in volatile compounds such as 2-hexenal, (*Z*)-2-heptenal, (*E*)-2-octenal, (*E*)-2-nonenal, (*E*,*Z*)-2,6-nonadienal and (*E*)-2-decenal associated with “green-like” aromas, rather than an increase in fruity and sweet aroma-impact compounds. Green rooibos extract powders would require storage at temperatures ≤ 30 °C and protection against moisture uptake to be chemically and physically shelf-stable and maintain their sensory profiles.

## 1. Introduction 

Rising global obesity and type 2 diabetes, which can be curbed only by addressing unhealthy diet and lifestyle as a long-term strategy [1], underpin initiatives, such as sugar tax on food and beverages to reduce sugar intake. As a consequence, reduction of their sugar content has become a priority [2]. The convergence of the concepts, sugar-free and functional ingredients with putative anti-diabetic/anti-obesity activities, allows the food industry to contribute actively to the promotion of health through the diet. Functional ready-to-drink (RTD) beverages, in particular, offer an excellent means of delivering nutrients and bioactive compounds in a convenient format to the consumer [3]. Challenges of this approach include degradation of bioactive/volatile compounds during food processing and storage [4] and a negative impact on taste [5].

A functional ingredient that gained interest in recent years due to its potential to modulate the metabolic syndrome is aspalathin-rich green rooibos extract (GRE) [6], shown to improve hyperglycaemia and HDL/LDL blood cholesterol profiles in non-human primates [7]. Aspalathin delivery in a RTD green rooibos iced tea presents a challenge as its stability is poor in solution, as shown by De Beer et al. [8]. In this study, the effect of the addition of common iced tea ingredients, ascorbic acid and citric acid, was also determined. Ascorbic acid enhanced the stability of aspalathin in the iced tea. The effect of citric acid, however, was ambiguous as it improved the stability of aspalathin in the RTD iced tea containing ‘fermented’ (oxidised) rooibos extract, while the opposite effect was observed when a green rooibos extract was used. During oxidation of rooibos plant material for production of the ‘fermented’ leaf product, substantial degradation of aspalathin occurs [9] accompanied by the formation of eriodictyol glucosides, followed by orientin and iso-orientin [10], dibenzofuran based dimers, followed by higher molecular weight browning products [11] and dihydrocaffeic acid [12]. Nothofagin, the 3-deoxy derivative of aspalathin and another bioactive dihydrochalcone [7], is by comparison present in low quantities in green rooibos extract. No information is available on its stability in solution or extract powder.

Another consideration is the sensory profile of a product, as it is usually regarded as one of the major drivers of consumer acceptance, even in the case of functional food and beverage products [13]. The aroma and flavour of foods and beverages have been shown to depend on the volatile compounds present. Storage can affect the volatile composition and in turn the sensory profile of teas [14,15].

In contrast to a RTD green rooibos iced tea, a powder iced tea formulation could potentially improve the physicochemical, chemical, and sensory stability of the product due to the low water activity (*a*_w_) of the powder. It is also possible to offer the consumer a formulated product in a convenient format such as in a single-serve sachet. However, mixing of ingredients can result in a range of physical and chemical interactions during storage, making it essential to consider these effects on final product quality. Compatibility of ingredients is not a given, as found for catechins in green tea powder [16] and phenolic compounds in green honeybush iced tea powders [17]. Both extract powders showed increased chemical and physical instability with the addition of ingredients such as citric acid and ascorbic acid.

The main objective of the current study was to determine the effect of ingredients, packaging, and storage conditions on the physical, chemical (volatile compounds and dihydrochalcones) and sensory shelf-life stability of a single-serve green rooibos iced tea powder containing inulin-microencapsulated GRE (IN50) as nutraceutical ingredient. The study revealed that to produce chemically and physically shelf-life stable powders, thermodynamically unstable crystalline ingredients should be avoided. Lower storage temperature and prevention of moisture uptake are required to maintain the original sensory and volatile compound profile.

## 2. Results and Discussion

### 2.1. Selection of Ingredients

Inulin, a prebiotic dietary fibre with a low kilojoule content [18], was used as an alternative microencapsulating agent to maltodextrin, the common carrier and bulking agent used by industry. Maltodextrin is, however, rapidly digested, contributing to an increase in postprandial blood glucose levels. It is therefore not a suitable carrier in the context of the anti-diabetic and anti-obesity functionality of aspalathin. Formulation of the green rooibos iced tea powders containing IN50 as the nutraceutical ingredient was further guided by the common food ingredients used in RTD rooibos iced teas [19], such as a sweetener, citric acid as an acidifier, and ascorbic acid as an antioxidant. The choice of xylitol, as an alternative sweetener to sucrose, was guided by its lower energy value compared to sucrose (10.0 vs. 16.7 kJ/g) and its sweetness that is nearly similar to that of sucrose [20]. A potential disadvantage of the use of xylitol could be gastrointestinal disturbances, however, at the level present in 330 mL of iced tea, this is not expected [21]. For comparative purposes, a mixture comprising of IN50 mixed with sugar (M1) was included as a control for a mixture of IN50 and xylitol (M2). The other mixtures contain IN50 and xylitol combined with either citric acid (M3), ascorbic acid (M4), or both (M5).

#### 2.1.1. Compatibility of Food Ingredients with IN50

Isothermal microcalorimetry was used to assess the compatibility of the ingredients in the powder mixtures as differences in the heat flow would indicate incompatibility and interaction between ingredients [22]. The difference between the average interaction heat flow values as obtained for the powder mixtures and the theoretical non-interaction curve obtained for the individual ingredients of the mixture was used to indicate possible incompatibilities (expressed as interaction average heat flow error) (Table 1). All of the interaction values were below 10 µW/g, thus indicating a low possibility of incompatibility and interaction of ingredients [22].

#### 2.1.2. Moisture Sorption Isotherms (MSIs)

**Figure 1:**
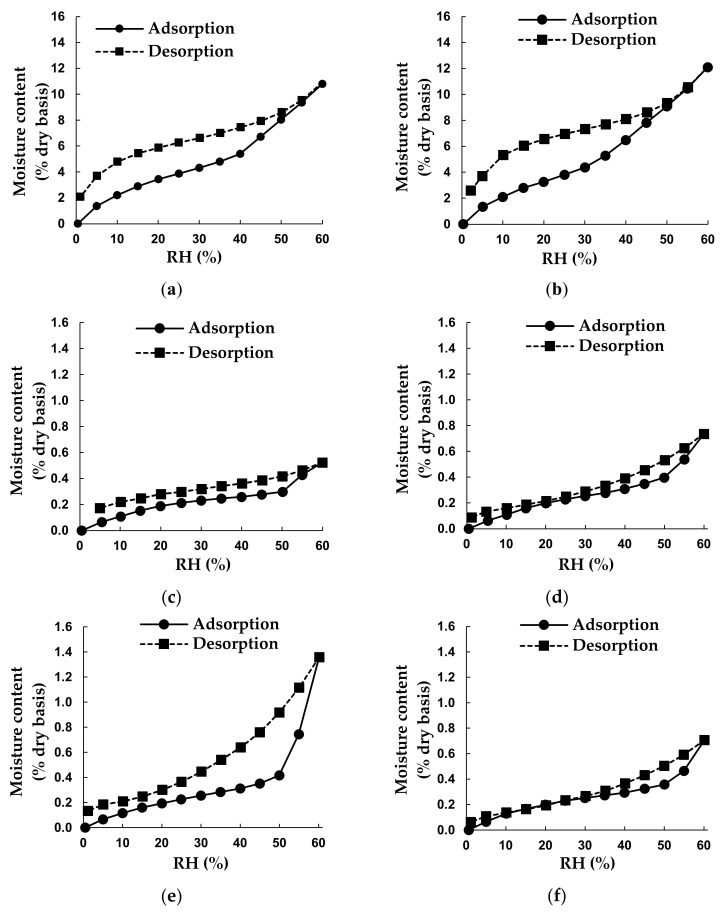

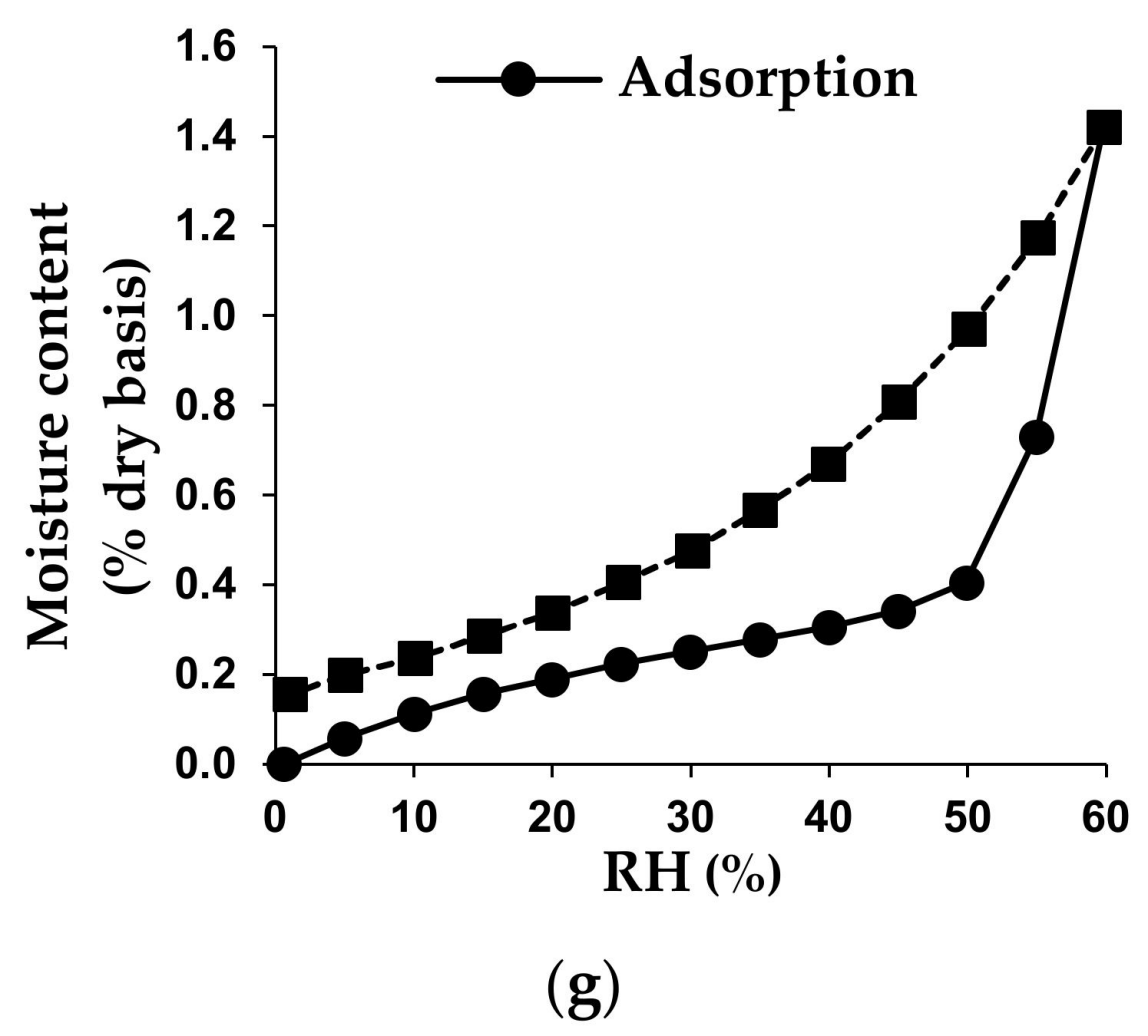
Moisture adsorption and desorption isotherms of green rooibos extract powder and powder mixtures at 25 °C: (**a**) green rooibos extract powder (GRE) (**b**) IN50 (green rooibos extract microencapsulated with inulin in 1:1 (m/m) ratio), (**c**) M1 (IN50 and sucrose), (**d**) M2 (IN50 and xylitol), (**e**) M3 (IN50, xylitol and citric acid), (**f**) M4 (IN50, xylitol and ascorbic acid) and (**g**) M5 (IN50, xylitol, ascorbic and citric acid).

The adsorption isotherms of the mixtures had the sigmoidal shape of type II isotherms, typical of food products [23] (Figure 1). Microencapsulation of GRE with inulin did not substantially change its MSI. The addition of deliquescent crystalline ingredients (sucrose, xylitol and acidifiers) to the amorphous IN50 did not change the isotherm type, but flattened the sigmoidal shape of the adsorption isotherm. The addition of crystalline sugar or xylitol to IN50 substantially reduced the moisture content (MC) of M1 and M2, up to 10 times at the same *a*_w_. The addition of ascorbic acid (M4) had little effect on the adsorption isotherm of M2, whereas the addition of citric acid (M3 and M5) resulted in a sharp increase above ca 50% relative humidity (RH), which is attributed to deliquescence [24]. The deliquescence RH (RH_0_) of citric acid monohydrate (78%) is the lowest of the different crystalline ingredients (sucrose, xylitol, and ascorbic acid), and in a mixture the RH_0_ would be less [24]. Crystalline substances have highly ordered structures that restrict molecular mobility and minimise free volume between molecules, allowing only minimal water uptake on the surface when kept below the RH_0_ [24], explaining the substantially lower MC values of M1–M5 compared to the amorphous IN50 (Table 1).

A hysteresis loop was evident for the MSI of each powder, most notably for IN50 and the mixtures containing citric acid monohydrate (M3 and M5). Mixing of IN50 with sucrose (M1) or xylitol (M2) decreased the hysteresis loop. Hysteresis arises from physical changes in the powders, which expose new sites able to adsorb moisture as the *a*_w_ decreases in the desorption curve [23]. Hysteresis can be used as a measure of food quality, as it indicates that exposure to moisture will result in permanent physical changes [23] and affect shelf-life stability. Thus, the addition of sucrose and xylitol will affect the shelf-life of IN50 positively, whereas the addition of citric acid will have a negative effect.

The moisture adsorption data were fitted to the Guggenheim–Anderson–de Boer (GAB) and Brunauer–Emmet–Teller (BET) adsorption models (Table 1). The BET model is only valid for 0.05 < *a*_w_ < 0.35 [23], but this model provided a better fit than the GAB model and was therefore fitted for *a*_w_ ≤ 0.3 (Table 1). The calculated monolayer moisture content values (*M*_0_) of the mixtures according to the BET model were between 0.22 and 0.27% (dry basis, d.b.), and were lower than their actual MC values (0.35–0.67%, d.b.). On the other hand, *M*_0_ values of GRE and IN50 were higher than their respective MC values. The same trends were observed for the GAB model. The *M*_0_ values provide an indication of water strongly bound to hydrophilic surface sites. For a shelf-stable product, the MC must be lower than the predicted *M*_0_ [25]. This is not the case for the mixtures M1 to M5, emphasising the importance of protection against changes in humidity during storage and handling of the mixtures.

### 2.2. Physical Changes in Powders during Storage

**Figure 2:**
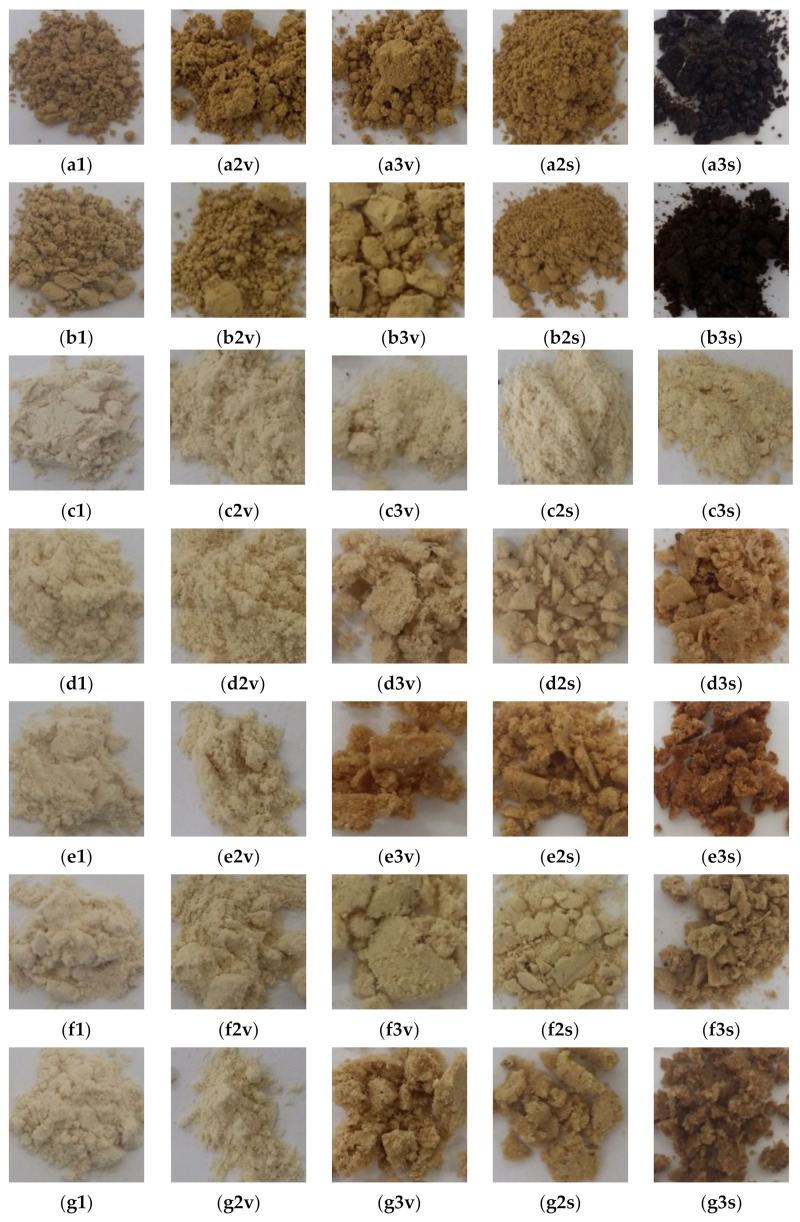
Change in visual appearance of the green rooibos extract powder and powder mixtures: (**a**) green rooibos extract (GRE), (**b**) GRE microencapsulated with inulin in 1:1 ratio (m/m) (IN50), (**c**) M1 (IN50 and sucrose), (**d**) M2 (IN50 and xylitol), (**e**) M3 (IN50, xylitol, and citric acid), (**f**) M4 (IN50, xylitol and ascorbic acid) and (**g**) M5 (IN50, xylitol, ascorbic acid, and citric acid), before (**1, a1–g1**) and after storage at 30 °C/65% RH (**2**) and 40 °C/65% RH (**3**) for 12 months in sealed glass vials (**v, a2v**–**g2v, a3v**–**g3v**) and 5 months in semi-permeable sachets (**s, a2s**–**g2s, a3s**–**g3s**).

**Figure 3:**
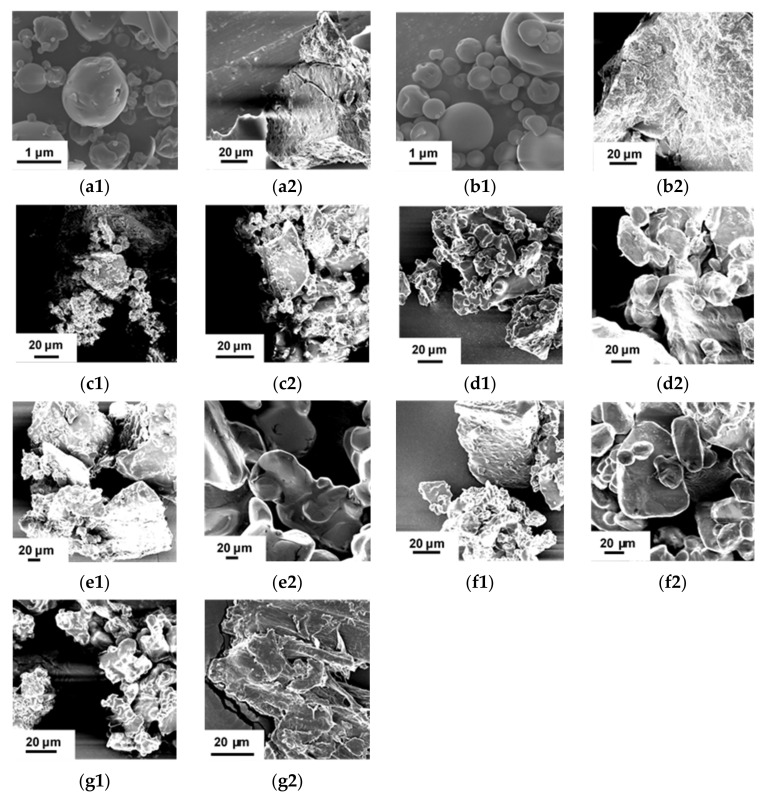
Scanning electron microscopy images of green rooibos extract powder and powder mixtures: (**a**) green rooibos extract (GRE), (**b**) GRE microencapsulated with inulin in 1:1 ratio (m/m) (IN50) and (**c**) M1 (IN50 and sucrose), (**d**) M2 (IN50 and xylitol), (**e**) M3 (IN50, xylitol, and citric acid) and (**f**) M4 (IN50, xylitol, and ascorbic acid), (**g**) M5 (IN50, xylitol, citric acid, and ascorbic acid) (**1, a1**–**g1**) before storage and (**2, a2**–**g2**) after storage at 40 °C/65% RH for 5 months in semi-permeable sachets.

**Figure 4:**
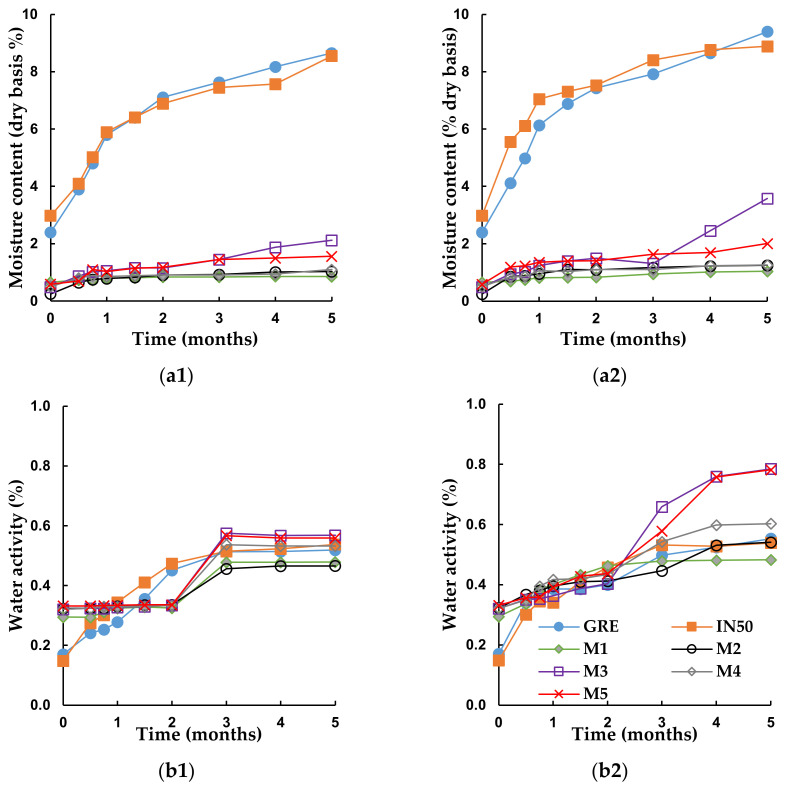
Moisture content (**a**) and water activity (**b**) of green rooibos extract powder and powder mixtures: green rooibos extract (GRE), GRE microencapsulated with inulin in 1:1 ratio (m/m) (IN50), M1 (IN50 and sucrose), M2 (IN50 and xylitol), M3 (IN50, xylitol, and citric acid), M4 (IN50, xylitol, and ascorbic acid) and M5 (IN50, xylitol, ascorbic acid, and citric acid), during storage at (**1, a1** and **b1**) 30 °C/65% RH and (**2, a2** and **b2**) 40 °C/65% RH for 5 months in semi-permeable sachets.

GRE showed clumping and slight discolouration after 12 months when stored at 30 and 40 °C in glass vials, despite protection against moisture uptake (Figure 2). Discolouration of GRE was extensive after 5 months in semi-permeable sachets at 40 °C/65% RH. Clumping of IN50 also occurred, but discolouration of the powder was slightly less, most likely due to the “dilution” effect of IN on the extract. The physical appearance of GRE and IN50 stored in the sachets changed from particulate to a sheet-like morphology (Figure 3), accompanied by a significant increase in MC (Figure 4), partially responsible for the drastic change in physical appearance. Additionally, IN50, but not GRE, showed evidence of crystallisation as evident from the sharp peaks on the X-ray powder diffractogram (XRPD, Supplementary material; Figure S1).

The other food ingredients affected the extent to which changes in the physical appearance of the powder occurred (Figure 2). Powders packed in the glass vials showed no or little obvious physical changes after 12 months, irrespective of storage temperature. Only M3 and M5, containing citric acid, showed some evidence of discolouration (browning) and clumping when stored at 40 °C. Except for a slight increase in the MC of the powder in the first month of storage (Supplementary material, Figure S2), no notable increase was observed, as would be expected for impermeable packaging material. The initial increase in MC was attributed to equilibration of the moisture trapped in the headspace of the vials and the powders.

The appearance of the powders packed in the semi-permeable sachets, especially when stored at 40 °C, changed substantially (Figure 2) with the formation of large and distorted particles (Figure 3) evident after 5 months. Replacement of sugar (M1) with xylitol (M2) resulted in clumping and slight browning, as well as a higher MC (1.04% vs. 1.25%) and *a*_w_ (0.48 vs. 0.54) after 5 months at 40 °C (Figure 4). This was to be expected, given their respective MSIs (Figure 1). The addition of citric acid resulted in more pronounced browning and clumping (M3 and M5). Not only were the prominent changes observed for M3 accelerated by the higher storage temperature, but its large hysteresis loop (Figure 1) and a significant increase in MC (from 0.49% to 3.58%) and *a*_w_ (0.32 to 0.79) (Figure 4) over 5 months at 40 °C indicated lower stability. The addition of ascorbic acid to the IN50 and xylitol mixture (M4) minimally decreased the slight discolouration observed for M2, thus offering some protection against oxidative browning. The final MC after 5 months at 40 °C were similar for M4 and M2 (1.25% vs. 1.26%, Figure 4) as predicted by very similar MSIs (Figure 1).

Plant extracts, such as GRE, are prone to oxidation and hydrolysis that result in colour changes [26]. Heinrich et al. [11] showed that aspalathin in solution forms a brown product under oxidative conditions, which was attributed to the formation of dibenzofurans. The colour change was most pronounced for GRE and IN50 consisting of 100% and 50% GRE, respectively. Oxidation reactions are often accelerated by the presence of moisture and temperature [26], which explains the extreme changes in colour and formation of clumps observed for the powders stored at 40 °C in the semi-permeable sachets compared to the sealed glass vials. However, the oxidation process could potentially be slowed by the addition of antioxidants, such as ascorbic acid, as shown by the slightly lower discolouration of M4 compared to M2. In contrast, Ortiz et al. [16] found that ascorbic acid increased the degradation of flavan-3-ols in green tea powder mixtures.

Furthermore, citric acid monohydrate is thermodynamically unstable at relatively low temperature and humidity (25 °C, < 65% RH) and is deliquescent in higher RH environments, where the transition from monohydrate to anhydrous citric acid occurs at 36.3 °C [27]. Thus, the addition of citric acid monohydrate would introduce moisture and result in higher instability as demonstrated for M3 and M5.

The observed visual changes of the powders stored in the sachets and at the higher temperature were highlighted by fusing, enlargement and more irregular shaped particles (Figure 3) attributed to hydration [28]. Combining and disappearance of peaks in the XRPD diffractograms of M1–M5 (Supplementary material; Figure S1) and crystallisation of IN50 in samples stored in the sachets at 40 °C further support physical changes. Additionally, changes in peak shape and intensities in the differential scanning calorimetry (DSC) thermograms (Supplementary material; Figure S3) indicate subtle changes in the molecular rearrangement of IN50 and crystal structures present in M1–M5. In the case of amorphous materials such as IN50, moisture acts as a plasticiser, leading to increased molecular mobility and lowering of the glass transition temperature (*T*_g_), resulting in crystallisation as excess adsorbed water is desorbed during storage [26]. In the case of formulations (M1–M5) containing deliquescent crystalline materials, the crystalline material can partially dissolve and re-crystallise, resulting in liquid bridges forming distorted crystal structures [29]. The rates of these effects are increased in the presence of moisture and at higher temperatures when more energy is available for molecular motion [24].

### 2.3. Chemical Stability and Kinetic Modelling of the Degradation of Dihydrochalcones

The change in aspalathin content as a function of storage time was modelled using the first order reaction kinetic model giving adjusted correlation coefficient (R^2^_adj_) values of between 0.70 and 0.98. This model is commonly utilised for the degradation of phenolic compounds, e.g., De Paepe et al. [30] and Beelders et al. [31]. The effects of storage temperature, packaging materials, microencapsulation, and ingredients on the stability of aspalathin were established according to reaction rate constants and extent of aspalathin degradation (Table 2). The small extent of aspalathin degradation (<10%) observed for GRE and IN50 at 30 °C, as well as IN50 at 40 °C, resulted in a poor fit of the kinetic model and values are therefore not reported.

The nothofagin content of the powders was also modelled using first order reaction kinetics. The decrease in nothofagin and the reaction rate constants (Supplementary material; Table S1) were comparable to those of aspalathin. The discussion will therefore be based on the results obtained for aspalathin.

The degradation rate constants and thus the extent of aspalathin degradation were higher at 40 °C than at 30 °C (Table 2). As an example, the extent of aspalathin degradation in the powders ranged from 6.66 to 50.6% and 9.67 to 90.3% for the samples stored in glass vials for 12 months at 30 °C and 40 °C, respectively. Furthermore, more extensive degradation was observed for aspalathin in powders stored in semi-permeable sachets compared to powders stored in vials for 5 months at 30 °C and 40 °C. The higher instability of aspalathin in the semi-permeable sachets agrees with the more drastic physical changes in the appearance of the powders (Figure 2). Exceptions were the aspalathin degradation rates for M4 and M5 stored at 30 °C and M5 stored at 40 °C. The discrepancies in reaction rates could be attributed to other variables having a larger effect than the increase in moisture due to the semi-permeability of the sachets.

In addition to the effects of temperature and packaging and thus moisture, the effects of the different ingredients are of interest as they substantially affected the physical stability of the formulations (Figure 2). In all cases, significantly less (*p* < 0.05) aspalathin degradation was observed for IN50 compared to GRE, except for powders stored in vials at 30 °C, which showed no significant (*p* ≥ 0.05) difference for these samples (Table 2). The extent of aspalathin degradation for GRE and IN50 was significantly (*p* < 0.05) lower compared to mixtures M1–M5, which contained crystalline ingredients. Furthermore, in all cases, except powders stored in sachets at 30 °C, the replacement of sucrose (M1) by xylitol (M2) resulted in significantly (*p* < 0.05) more aspalathin degradation. Aspalathin degradation in sachets stored at 40 °C was 39.8% for M1 and 61.5% for M2 after 5 months. Storage of M1 and M2 in the vials at 40 °C resulted in 34.0 and 40.8% aspalathin degradation after 5 months. The reaction rate constants showed similar trends. The addition of citric acid to the IN50 and xylitol powder formulation (M3) resulted in increased aspalathin instability in all cases, except during storage in the sachets at 40 °C, as shown by the extent of aspalathin degradation and reaction rate constants. This observed exception could be due to unexpected evaporation of moisture as shown by a slight decrease in the MC of the powders at 3 months (Figure 4); however, the MC did increase rapidly again after this. The addition of ascorbic acid (M4) showed similar results with decreased aspalathin stability except for the powders stored in the vials at 30 °C, which showed no significant (*p* ≥ 0.05) difference to M2. Generally, as expected, the combination of the citric and ascorbic acid (M5) also showed decreased aspalathin stability compared to M2.

The increase in aspalathin degradation with an increase in temperature is typical for phenolics as previously shown for dihydrochalcones in cloudy apple juice [30], epigallocatechin gallate (EGCG) powder [32], and xanthones and benzophenones in honeybush plant material [31]. The general higher instability of aspalathin in the powders stored in the semi-permeable sachets could be due to their higher MC, as confirmed by increases in MC and *a*_w_ for all the formulations during storage (Figure 4). The detrimental effect of high RH on phenolic stability and specifically aspalathin is not uncommon and have been shown for green tea powders [16] and flavone *C*-glycosides [26]. 

The effect of microencapsulation and the addition of crystalline ingredients become more complicated to understand as also indicated by Ortiz and colleagues [16,33]. Generally, higher physical and chemical instability have been reported for blends of deliquescent crystalline and amorphous ingredients compared to individual amorphous components [34]. Ortiz and colleagues [16,33] found that the addition of citric acid to amorphous green tea powders resulted in a decrease in physical and chemical stability. Similarly, De Beer et al. [17] found a decrease in xanthone stability with the addition of ascorbic and citric acid to honeybush powders. Generally, the trend in the current study also seems to indicate that the addition of these acids results in higher aspalathin instability, however; this was not always the case. Citric acid monohydrate, as used in the current study, tends to dehydrate under various storage conditions [27]. In the sealed glass vials, this dehydration most probably resulted in the wetting of the powders, explaining the decreased stability of M3. However, in the sachets at 40 °C where the powders were already exposed to moisture and large amounts of water was absorbed from the storage cabinet environment (65% RH), the extra moisture produced by the citric acid was probably negligible and affected the aspalathin stability to a lesser extent. Ascorbic acid is generally added to food formulations as an antioxidant to decrease chemical degradation as shown for aspalathin in RTD iced tea [35]. However, the results of the present study and those by Ortiz and colleagues [16,33] showed that the effect of ascorbic acid on powder stability is more complex.

### 2.4. Change in the Sensory Profile of M3 during Storage

Given the more severe physicochemical changes observed for M3 during storage (Figure 2 and Table 2) compared to the other mixtures, it was of interest to determine whether noticeable sensory changes also took place in this mixture. For a quick screening, samples were stored for 1 month at 30 °C/65% RH and 40 °C/65% RH in sealed vials and semi-permeable sachets and reconstituted in deionised water (63 g/L) to beverage strength before sensory testing.

**Figure 5:**
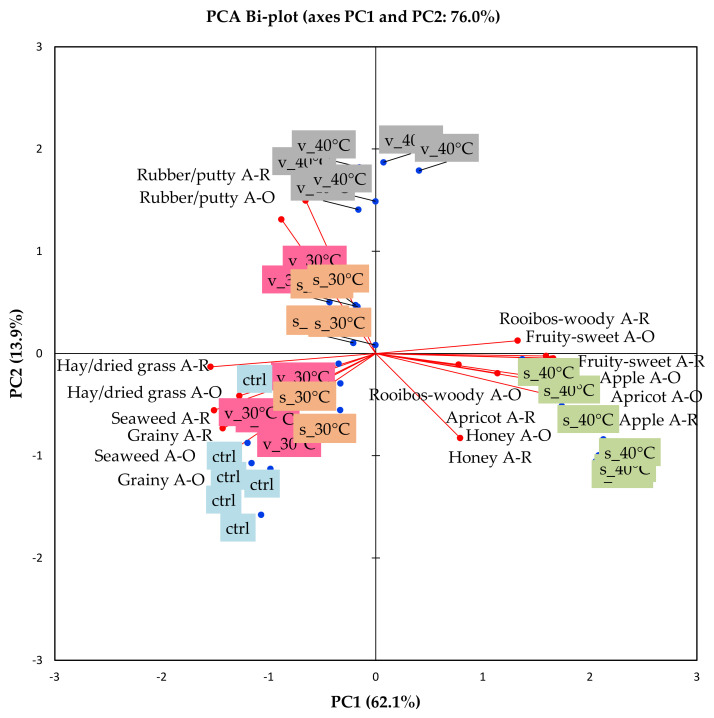
Principal component analysis (PCA) bi-plot showing the position of selected sensory attributes (orthonasal (A-O) and retronasal (A-R) aroma) in relation to M3 reconstituted in water to beverage strength (green rooibos extract microencapsulated with inulin in 1:1 ratio (m/m), xylitol, and citric acid), before storage (control, ctrl) and stored at 30 °C/65% RH and 40 °C/65% RH for 1 month in sealed glass vials (**v**) and semi-permeable sachets (**s**).

**Figure 6:**
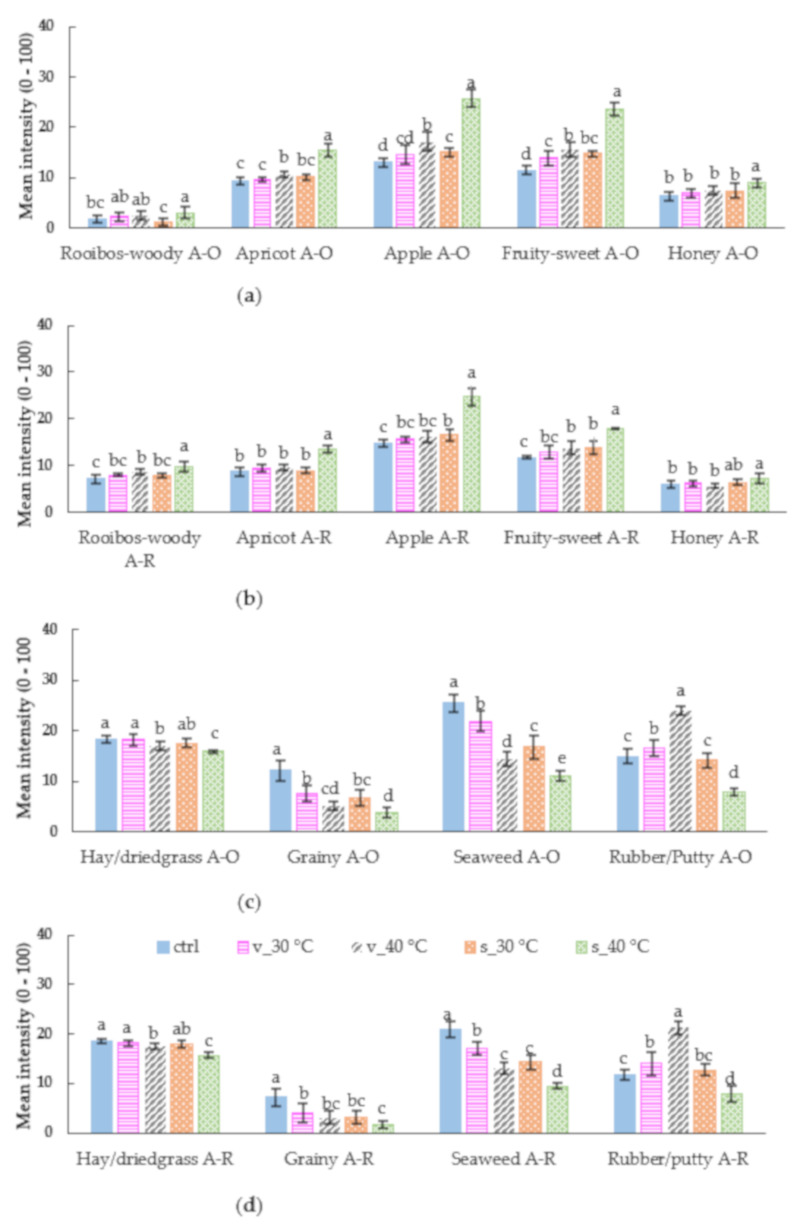
Mean intensities of the positive (**a**) orthonasal (A-O) and (**b**) retronasal (A-R) aroma attributes, as well the negative (**c**) orthonasal (A-O) and (**d**) retronasal (A-R) aroma attributes for M3 reconstituted in water to beverage strength (green rooibos extract microencapsulated with inulin in 1:1 ratio (m/m), xylitol, and citric acid), before storage (control, ctrl) and stored for 1 month at 30 °C/65% RH and 40 °C/65% RH in sealed glass vials (v) and semi-permeable sachets (s). Means with different letters, within a sensory attribute, differ significantly (*p* ≤ 0.05).

**Figure 7:**
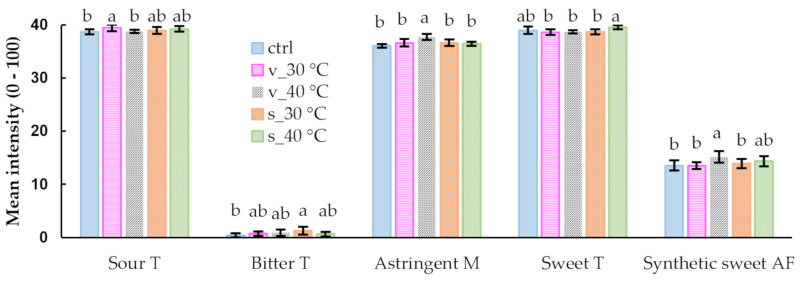
Mean intensities of the taste (T), mouthfeel (M) and aftertaste (AF) attributes for M3 reconstituted in water to beverage (green rooibos extract microencapsulated with inulin in 1:1 ratio (m/m), xylitol, and citric acid), before storage (control, ctrl) and stored for 1 month at 30 °C/65% RH and 40 °C/65% RH in sealed glass vials (v) and semi-permeable sachets (s). Means with different letters, within a sensory attribute, differ significantly (*p* ≤ 0.05).

The principal component analysis (PCA) bi-plot (Figure 5) shows the association of the samples with the aroma attributes tested orthonasally and retronasally. The mean intensities of the aroma attributes, as well as that of taste, astringency (mouthfeel), and ‘synthetic’ sweet aftertaste, are shown in Figure 6 and Figure 7. The aroma attributes followed similar trends as previously found for rooibos infusions, although the infusions were evaluated hot [36,37], i.e., all the attributes were perceived at higher intensities when evaluated orthonasally compared to retronasally, except for ‘rooibos-woody’. Therefore, only orthonasal attributes will be discussed further.

The first two principal components (PC1 and PC2) of the PCA bi-plot (Figure 5) explain 76.0% of the variance. M3 stored in sachets at 40 °C are separated from the other storage treatments on PC1. This sample, associated more with the aroma attributes ‘rooibos-woody’, ‘honey’, ‘apple’, ‘apricot’, and ‘fruity-sweet’ (Figure 5). The intensities of all the aroma attributes of this sample were significantly higher (*p* < 0.05) than that of the other storage treatments (Figure 6). These aroma attributes are all typical of ‘fermented’ rooibos tea [36], i.e., traditional rooibos tea oxidised by wetting, bruising and drying. However, in traditional rooibos tea, the intensity of the ‘rooibos-woody’ aroma is usually much more prominent [38].

The other storage treatments on the left of PC1 were either associated with ‘rubber/putty’ (M3 stored in vials at 40 °C) or with ‘hay/dried grass’, ‘seaweed’, and ‘grainy’ (Figure 5). The intensities of the attribute ‘hay/dried grass’ were quite low and reasonably similar for all storage treatments, except for M3 stored in sachets at 40 °C, which resulted in a significantly lower intensity (*p* < 0.05) for this attribute (Figure 6). The intensity of ‘hay/dried grass’ in all the experimental treatments of the storage experiment was on par with that of traditional ‘fermented’ rooibos tea [38].

The attributes ‘seaweed’, ‘grainy’, and ‘rubber/putty’ are atypical for ‘fermented’ rooibos tea, however, they can be regarded as an element of brewed green rooibos tea, as previously demonstrated [38]. In the current experiment, storage of M3 in the vials at 40 °C resulted in a significantly (*p* < 0.05) higher intensity for the putty-like aroma attribute (Figure 6); however, this attribute was also quite perceptible in regular brewed green rooibos tea [38]. Similarly, the aroma attributes, ‘seaweed’ and ‘grainy’ can be associated with green rooibos iced tea and brews, as shown in this study (Figure 6), as well as previous research [38], respectively. In the current research, the intensities of both these attributes were significantly higher (*p* < 0.05) in the control than the other storage treatments.

The perceived increase (*p* < 0.05) in astringency for the samples stored in the vials at 40 °C could be due to various reasons, where differences in the changes of the phenolic profile of the samples could be responsible [39]. An increase in astringency could indicate formation of oxidation products with greater ability to bind to salivary proteins. However, the volatile composition of the samples also changed (as discussed later) and the cross-modal effect of volatiles on astringency and taste perceptions may therefore also play a role as demonstrated for wine and model solutions [40,41]. Labbe et al. [42] demonstrated that ‘sweet’ aroma compounds at a subthreshold level can significantly modulate sweet taste perception of a sucrose solution.

The change in the sensory profile of M3 during storage was also accompanied by a change in the colour of the powders reconstituted in water for consumption (Table 3). The pH values of all the solutions were similar (pH 2.88–2.89). The changes in L*, a*, b*, C* and h values of the solutions were consistent with the browning observed for the powders, associated with the oxidation of rooibos phenolic compounds [11].

### 2.5. Change in the Volatile Profile of M3 during Storage

Given the significant changes in the sensory profile observed for M3 (Figure 5 and Figure 6) during storage at 40 °C/65% RH, it was of interest to determine whether there were changes in the volatile profile and how this correlated to the sensory profile. For a quick screening, samples were stored for 1 month at 40 °C/65% RH in sealed vials and semi-permeable sachets and reconstituted samples (in deionised water at 63 g/L) analysed by gas chromatography time-of-flight mass spectrometry (GC-TOF-MS).

**Figure 8:**
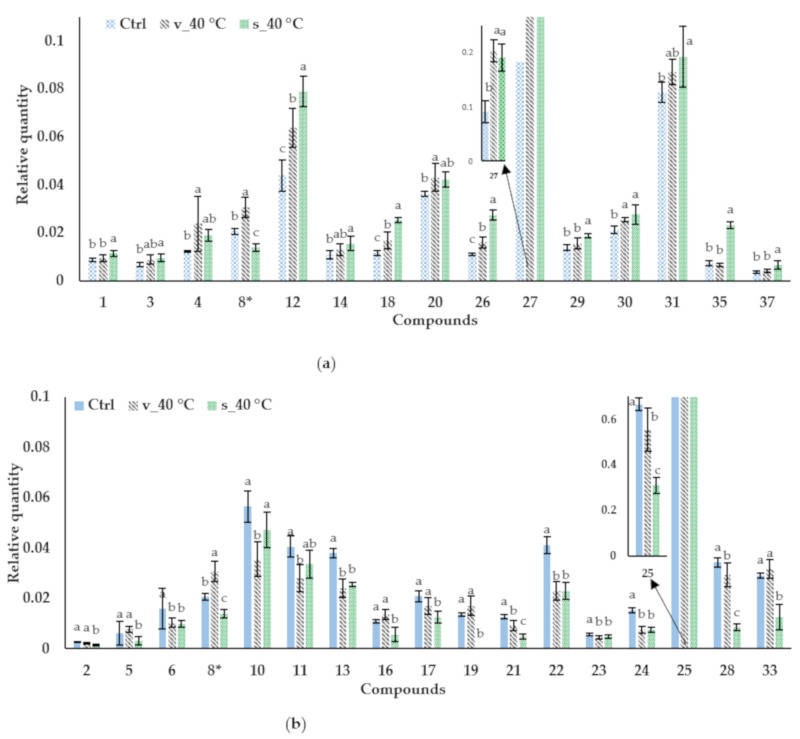
Mean relative quantities (calculated as peak area of compound divided by that of internal standard) of identified volatile compounds for M3 reconstituted in water to beverage strength (green rooibos extract microencapsulated with inulin in 1:1 ratio (m/m), xylitol, and citric acid), before storage (control, ctrl) and after 40 °C/65% RH in sealed glass vials (v) and semi-permeable sachets (s) where (**a**) shows compounds that increased and (**b**) decreased significantly after storage. Means with different letters for each compound differ significantly (*p* ≤ 0.05). * Compound **8** increased and decreased significantly after storage in the vials and sachets, respectively and is present in graphs (**a**,**b**).

Table 4 shows volatile compounds identified in M3 before and after storage, their calculated and literature retention indices and aroma descriptors. Thirty-seven compounds were identified, belonging to the aldehyde, terpene, terpenoid, ester, furan, ketone, alcohol, enone, aromatic hydrocarbon, and organic acid classes, amongst others. The bulk of the compounds identified have been detected previously in rooibos [43,44,45]. 6-Methyl-5-hepten-2-one (**2**), 6-methyl-3,5-heptadien-2-one (**20**), (*E*)-β-damascenone (**25**), geranyl acetone (**27**) [44], and octanoic acid (**31**) [45] were found to be present in higher quantities compared to other compounds in fermented rooibos tea, as is the case in the present study (Figure 8).

Of the 37 compounds identified, 31 increased (Figure 8a) or decreased (Figure 8b) significantly (*p* < 0.05) during storage in the sachets or vials or both and will be discussed further. For several of the compounds, including 6-methyl-5-hepten-2-one (**12**), 1-octanol (**18**), hexanoic acid (**26**), 2,5,5,8α-tetramethyl-3,5,6,8α-tetrahydro-2H-chromene (**21**), (*E*)-β-damascenone (**25**), and α-calacorene (**28**), the changes observed were larger for the samples stored in the sachets than in the vials, where for the remaining compounds there was no significant difference between the sachets and vials.

Generally, the acids, hexanoic acid (**26**), octanoic acid (**31**), heptanoic acid (**30**), and hexadecanoic acid (**37**), and the alcohols, 1-octen-3-ol (**14**), and 1-octanol (**18**), increased during storage, except 1-nonanol (**23**), which showed a slight decrease. The alcohols and acids are associated with a wide variety of aroma descriptors (Table 4).

The aldehydes, 2-hexenal (**5**), (*Z*)-2-heptenal (**11**), (*E*)-2-octenal (**13**), (*E*)-2-nonenal (**16**), (*E*,*Z*)-2,6-nonadienal (**19**), (*E*)-2-decenal (**22**), and 2-undecenal (**24**), except hexanal (**1**), decreased. These aldehydes are generally associated with “green” aromas.

The changes in the terpenes, ketones and enone, generally more associated with the typical aroma of fermented rooibos (floral, woody, fruity), were varied. The terpenoid, limonene (**4**), and terpenone, geranyl acetone (**27**), increased, whereas linalool (**17**) and the other terpenes, β-myrcene (**2**) and α-calacorene (**28**), decreased. The ketone, 1-octen-3-one (**10**), and enone, (*E*)-β-damascenone (**25**), decreased, whereas the other ketones, 6-methyl-5-hepten-2-one (**12**) and 6-methyl-3,5-heptadien-2-one (**20**), all major compounds in rooibos infusions and in the current samples, increased.

The degradation and loss of volatile compounds observed during storage are not uncommon and the corresponding impact on flavour stability of tea-based beverages is a long existing concern [46]. The oxidation of electron-rich volatile compounds, such as terpenes and aldehydes, is likely to occur upon contact with air [47,48], possibly accounting for the decreases observed for linalool, β-myrcene, and α-calacorene and the majority of the aldehydes. The free radical chain process of their oxidation can be accelerated by heat, light, and moisture [47,48]. Furthermore, volatile compounds could be lost via evaporation in the semi-permeable packaging, explaining the apparent higher degradation of certain compounds in the sachets than in the vials.

The formation and increase of volatile compounds during storage can be either directly via oxidative degradation of precursor compounds, such as carbohydrates, carotenoids, amino acids, and fatty acids that are present or, further metabolism of primary fatty acid-derived molecules [49]. The increases observed for limonene and geranyl acetate were likely due to condensation of common precursors, isopentenyl diphosphate and dimethylallyl diphosphate, produced by carbohydrates present and acetyl coenzyme A [50,51]. However, in the case of a beverage prepared from an aqueous extract, such as GRE, compared to plant material, some of the more lipophilic/hydrophobic precursor molecules may not be present or present in very low quantities. These limitations could clarify why no increases were observed for linalool, and (*E*)-β-damascenone, with floral/fruity aroma descriptors, which were found to increase with DSA. However, it could also be that the rate of oxidative degradation, instead of formation, of these compounds was favoured under the storage conditions investigated, as is possibly also the case for the decrease in β-myrcene and α-calacorene. Furthermore, primary fatty acid-derived aldehydes (such as 2-hexenal) can be further auto-oxidised to their corresponding alcohols by a free radical chain process [47], which could explain the decrease and increase in certain aldehydes and alcohols, respectively.

**Figure 9:**
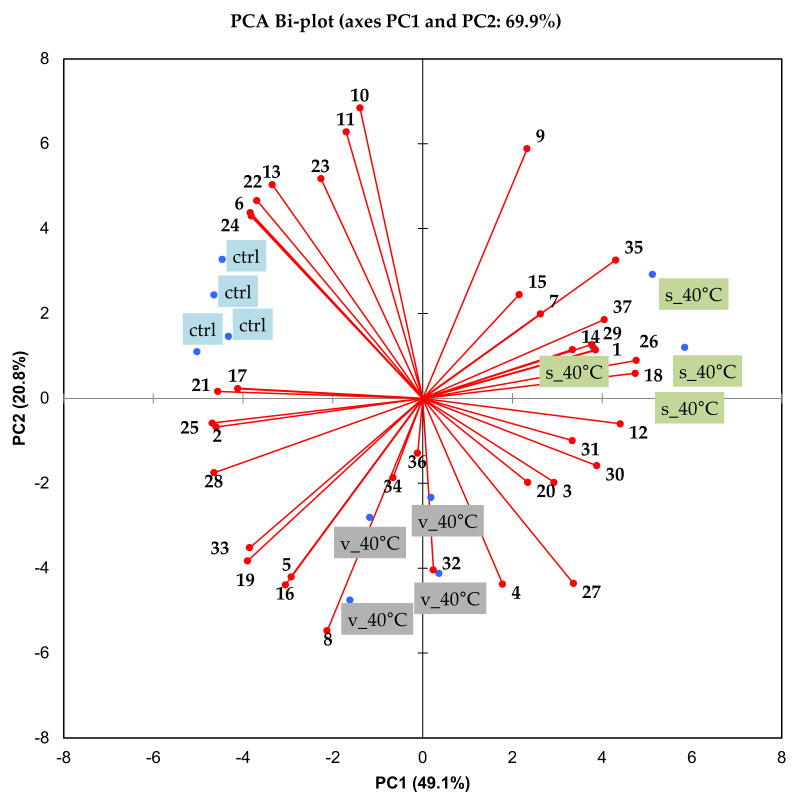
Principal component analysis (PCA) bi-plot showing the position of identified volatile compounds in relation to M3 reconstituted in water to beverage strength (green rooibos extract microencapsulated with inulin in 1:1 ratio (m/m), xylitol, and citric acid), before storage (control, ctrl) and stored at 40 °C/65% RH for 1 month in sealed glass vials (**v**) and semi-permeable sachets (**s**).

The PCA bi-plot in Figure 9 shows the association of samples with the volatile compounds. The first two principal components (PC1 and PC2) explain 69.9% of the variance. Samples stored in the sachets at 40 °C are separated from the control on PC1, while samples stored in vials at 40 °C are separated from the control on PC2. The control samples are associated with the volatile compounds 2,5,5,8α-tetramethyl-3,5,6,8α-tetrahydro-2H-chromene (**21**), linalool (**17**), 2-undecenal (**24**), 2-pentylfuran (**6**), (*E*)-2-decenal (**22**), (*E*)-2-octenal (**13**), and 1-nonanol (**23**). Compounds **6**, **13** and **22** are described as having “green” type aromas, fitting the sensory descriptions of “dried grass” and “seaweed” used to describe the control samples. Compounds **17**, **23**, and **24** are described as having “floral” and “fruity” aromas, which were detected by DSA in the control samples, but at lower intensities.

The samples stored in the vials at 40 °C were associated with limonene (**4**), 2-hexenal (**5**), cymene (**8**), (*E*)-2-nonenal (**16**), and nonanoic acid (**32**). The samples stored in the sachets at 40 °C were associated with hexanal (**1**), 6-methyl-5-hepten-2-one (**12**), 1-octen-3-one (**14**), 1-octanol (**18**), hexanoic acid (**26**), 3-(2,6,6-trimethyl-1-cyclohexen-1-yl-2-propenal) (**29**), 3,5-di-tert-butylphenol (**35**), and hexadecanoic acid (**37**). No clear correlation could be drawn between the aroma characteristics and volatiles associated with a specific sample after storage since none of the identified compounds matched the aroma attributes described in the DSA. However, various substances act synergistically to form the aroma of beverages [46], where each compound also has a specific odour threshold concentration. Du Preez et al. [44] found that for rooibos aroma attributes such as ‘fynbos-floral’ and ‘apricot’ no single aroma chemical compound fully represented these aromas. The complicated nature of the relationship of aroma and volatile compounds and the fact that only compounds that could be identified by comparing retention indices were selected, limit the possibility of linking specific volatile compounds to a specific sensory attribute.

## 3. Materials and Methods

### 3.1. Materials and Chemicals

GRE containing 14.64% aspalathin, 1.42% nothofagin, 0.50% glucose, and 1.92% pinitol (d.b.) was supplied by a commercial extract manufacturer. Inulin (21–26 degree of polymerisation), extracted from chicory root (*Cichorium intybus* var. sativum) was supplied by Savannah Fine Chemicals Ltd. (Cape Town, South Africa) and xylitol by Warren Chemicals (Cape Town, South Africa). Food grade citric acid monohydrate and ascorbic acid were obtained from Cape Food Ingredients (Westlake, South Africa), and sucrose (white sugar) was purchased from a local supermarket. High-performance liquid chromatography (HPLC) grade acetonitrile and analytical grade chemicals were obtained from Merck (Darmstadt, Germany). Aspalathin was supplied by the South African Medical Research Council of South Africa (Cape Town, South Africa, > 95% purity by HPLC-mass spectroscopy and -diode array detection (DAD)). Sodium chloride (NaCl), β-phenylethyl butyrate and a mixture of linear hydrocarbons (C7–C40) were obtained from Sigma Aldrich, St. Louis, MO, USA.

### 3.2. Spray-Drying and Microencapsulation

Spray-drying of GRE and a GRE-inulin mixture in a 1:1 (m/m) ratio (IN50) was done using a Büchi B-290 mini spray-drier (Büchi Labortechnik AG, Flawil, Switzerland), employing conditions described by Miller et al. [52].

### 3.3. Preparation of Green Rooibos Powders

In addition to the controls (GRE and IN50), five mixtures (M1 to M5) containing different combinations of IN50 and other ingredients were also investigated (Table 5). The ratios of the various ingredients were based on an RTD iced tea formulation mixture containing 1.75 g/L rooibos extract, 60 g/L sucrose, 1.2 g/L citric acid, and 0.2 g/L ascorbic acid. The amount of xylitol varied slightly between M2 and M5, to accommodate the addition of citric acid and ascorbic acid, while maintaining a constant final concentration of 63.17 g/L powder mixture and 3.5 g/L IN50 (equalling 1.75 g/L GRE) for all powders.

The sugar, citric acid, and ascorbic acid were ground separately to a fine powder (Fritsch GmbH Pulverisette ball mill, Idar-Oberstein, Germany) and sieved to ensure a small particle size (<210 µm; Endecotts Ltd., London, England). IN50 and the food ingredients were mixed by a slow tumbling action, using a glass mixing vessel attached to the rotary driving mechanism of a rotary evaporator (Büchi Labortechnik AG, Flawil, Switzerland.

### 3.4. Storage Stability Testing and Kinetic Modelling of Aspalathin and Nothofagin Degradation

Aliquots (ca 500 mg) of the different powders, representing the treatments (n = 7), were weighed into sachets and glass vials, representing semi-permeable and impermeable packing. The sachets, produced from 12 µm metalised polyethylene terephthalate (MET/PET) film and a 50 µm linear low-density polyethylene (LLDPE) film attached with a layer of adhesive, allowed moisture uptake (0.4% transmission of moisture vapour measured as g/m^2^ in 24 h) [53]. The amber glass vials (5 mL) were tightly sealed using screw caps with polytetrafluoroethylene (PTFE) lined septa, thus simulating impermeable packaging as would be provided by three-layer (e.g., polyester film/aluminium foil/LDPE) sachets with minimal to no transmission of moisture vapour (0.01%) [53]. Samples were stored at 30 °C and 40 °C in stability cabinets with the RH controlled at 65% (SMC Scientific Manufacturing cc., Table View, South Africa). The viewing windows of both cabinets were blocked with non-transparent material to eliminate light. All powders were sampled nine times during storage, starting at *t* = 0. Sachets containing the powders were removed weekly during the first month and thereafter monthly for up to 5 months. Vials containing the same powders were removed monthly up to 6 months and every 3 months thereafter up to 12 months. The shorter storage period of 5 months for the sachets was selected due to drastic changes in the physical appearance after this time. Following removal from storage, aliquots were taken for various analyses.

The aspalathin and nothofagin content of the powders, reconstituted in water, were determined by HPLC-DAD [54]. The first order reaction kinetic model was fitted to the data to calculate the reaction rate constant [55] for the degradation of these compounds in GRE as affected by different storage conditions and ingredients.

### 3.5. Physicochemical Analysis

MSIs of GRE, IN50 and other powder mixtures (M1 to M5) were determined before storage, while their MC and *a*_w_ were determined before and during storage. MC and *a*_w_ measurements during storage were performed on a pooled sample (n = 3, replicate samples) due to the limited amount of sample. Isothermal microcalorimetry was performed to detect whether IN50 was incompatible with the other ingredients, an indication of potential instability. Particle morphology of the mixtures before and after storage was determined by scanning electron microscopy (SEM) imaging. Experimental details are described in Miller et al. [52]. Other analyses were XRPD and DSC of the ingredients and mixtures before and after storage. Experimental details are described in Human et al. [56]

### 3.6. Descriptive Sensory Analysis (DSA), PH, and Colour of Reconstituted Beverages

The reference samples for panel training included extracts prepared from green rooibos (Rooibos Ltd., Clanwilliam, South Africa), semi-‘fermented’ rooibos (shredded green rooibos plant material, ‘fermented’ for 1 h at 37 °C and dried at 40 °C for 16 h) and ‘fermented’ rooibos material (Rooibos Ltd., Clanwilliam, South Africa). The plant material was extracted with deionised water (3 g/mL) at 93 °C for 40 min, cooled and served at ambient temperature (±21 °C). The reference samples were chosen to represent the full spectrum of aroma attributes associated with the reconstituted green rooibos powders and an infusion of oxidised rooibos. Additionally, xylitol (58 g/L) and citric acid (1.2 g/L) were used as reference standards for ‘synthetic’ sweet aftertaste and sour taste, respectively.

M3 was selected for sensory analysis, given the significant changes observed during storage at 40 °C/65% RH (Figure 5 and Figure 6). Six replicates of M3 was prepared and aliquots of each replicate were divided and packed in sachets and vials, which were then stored at 30 °C and 40 °C at 65% RH for 1 month as described in Section 3.4 (i.e., six random replicates for each of the four treatment combinations). The control treatment was M3 freshly prepared on the day of testing. The samples were reconstituted in deionised water at 63 g/L and aliquots of ca 70 mL served in standard ISO wine tasting glasses covered with a plastic lid at ambient temperature (±21 °C).

The panel consisted of 12 female judges with extensive training in DSA. During five one-hour training sessions, the panel came to a consensus on the range of orthonasal aroma, retronasal aroma (flavour), taste, mouthfeel, and aftertaste descriptors (Supplementary material, Table S2). The rooibos lexicon developed by Koch et al. [37] was used as a basis and definitions were adjusted where necessary.

The full sample set (n = 5) was tested during each session, with two sessions per day over 3 days to analyse all replicate samples. Testing was conducted in individual tasting booths on samples labelled with three-digit codes and presented in a completely randomised order, specific to each panellist. Water and dried apple rings were provided as a palate cleanser between samples. The relevant attributes were scored on an unstructured scale from 0 (not detected) to 100 (extremely high intensity). Scores were captured with Compusense^®^ *five* software (Compusense, Guelph, Canada).

The pH of each solution was measured (Crison GLP 21 pH meter, Crison Instruments SA, Alella, Spain). CIE L*a*b* objective colour measurements of the solutions were performed using a Konica Minolta CM-5 spectrophotometer (Osaka, Japan) in transmittance mode and 10 mm polystyrene cuvettes. The instrument was calibrated using a black calibration plate and a cuvette filled with deionised water. The ΔE of samples before and after storage was calculated as follows (Equation (1)):

(1) $$∆E=\sqrt{{\left(L_{2}^{*}-L_{1}^{*}\right)}^{2}+{\left(a_{2}^{*}-a_{1}^{*}\right)}^{2}+{\left(b_{2}^{*}-b_{1}^{*}\right)}^{2}}$$

where subscripts 1 and 2 refer to values before and after storage, respectively.

### 3.7. GC-TOF-MS Analysis of Reconstituted Beverages

Aliquots of four replicates of M3 were packed in sachets and vials and stored at 40 °C and 65% RH for 1 month as described in Section 3.4. All samples were reconstituted in deionised water to 63 g/L and frozen until analysis.

#### 3.7.1. Headspace-Solid Phase Micro-Extraction (HS-SPME) Procedure

The samples were defrosted at room temperature and 10 mL of tea solution was placed in a 20 mL headspace vial containing 2 g NaCl together with the internal standard (IS) at 100 µg/L (10 µL of β-phenylethyl butyrate, 100 mg/L in ethanol). An SPME fibre (50/30 µm PDMS/CAR/DVB) from Supelco (Bellefonte, PA, USA) was used for extraction of volatiles from the headspace of the sample. The fibre was conditioned according to the specifications of the manufacturer before use. The samples were pre-incubated at 65 °C for 5 min and HS-SPME extraction was performed for 45 min at 65 °C with agitation at 100 rpm. The analytes were subsequently desorbed in the GC injector at 240 °C for 5 min.

#### 3.7.2. GC-TOF-MS Conditions

Analyses were carried out on a LECO GC-TOF-MS instrument (LECO Corp., St. Joseph, MI, USA) consisting of a TOF-MS, an Agilent 7890B GC (Agilent Technologies, Palo Alto, CA, USA) equipped with a split/splitless injector and a Gerstel MPS multi-purpose sampler (Gerstel, Mulheim ad Ruhr, Germany). Helium was used as a carrier gas for the GC at a constant flow of 1.0 mL/min. The injector was operated at 240 °C in splitless mode for SPME injections (splitless for 2 min, thereafter 1:30 split ratio). The column used was a polar 30 m × 0.25 mm ID (internal diameter) × 0.25 μm d_f_ (film thickness) ZB-FFAP column (Phenomenex, Torrance, CA, USA). The GC oven was programmed from 40 °C, held for 2 min, ramped at 5 °C/min to 240 °C and held for 2 min. The transfer line and ion source were set to 250 °C and 200 °C, respectively, and the detector voltage was 1650 V. Data were acquired at a rate of 13.3 spectra/s with a mass scan range of 40–350 amu.

#### 3.7.3. Data Processing

Data processing was performed using ChromaTOF^®^-GC-TOF-MS software (LECO Corp., version 5.32.12.0) incorporating an algorithm for peak deconvolution. Compounds were tentatively identified using an MS library search matching against the NIST 11 MS library (National Institute of Standards and Technology, Gaithersburg, MD, USA) and comparison of calculated linear retention indices with literature values. Data from analysis of a mixture of C7–C40 linear hydrocarbons were used for calculation of the retention indices. Peak areas for the peaks of interest were calculated by the data processing software based on the area of the base peak ion in the mass spectrum recorded for the compound. Relative concentrations were manually calculated and reported as the integrated peak area of the compound divided by the integrated peak area of the IS as described in Pati et al. [57].

### 3.8. Statistical Analyses

#### 3.8.1. Rooibos Powder Stability Data

The experimental design was completely random with three independently replicated experiments for each treatment (n = 7) in the initial storage experiment. Univariate analysis of variance (ANOVA) was performed on all observed variables to compare treatments, using the GLM (General Linear Models) procedure of SAS software (Version 9.4; SAS Institute Inc., Cary, NC, USA).

Non-linear regression analysis with time as the independent variable was performed to describe aspalathin and nothofagin degradation over time, using the NLIN procedure of SAS. The first order reaction kinetic model was fitted for each experimental replicate. The regression parameters obtained were used as input for ANOVA to test for the effect of treatment on the model parameters. Data were tested for normality using the Shapiro-Wilk test. Fisher’s least significant difference (LSD) was calculated at the 5% level to compare means and a *p*-value < 0.05 was considered significant.

#### 3.8.2. DSA and GC-TOF-MS Data

Panel performance was monitored by applying PanelCheck software (Version 1.4.0; Nofima, Ås, Norway) to the data obtained. The data were pre-processed according to the model suggested by Næs et al. [58] that includes evaluation of panellists, replicate, sample, and interaction effects. Outliers were removed where necessary following assessment of normality of the standardised residuals using the Shapiro-Wilk test.

ANOVA was conducted on the data representing five treatments (M3 stored under different conditions), 12 judges and six replicate sessions. PCA, based on the correlation matrix, was also conducted using XLStat software (Addinsoft, Paris, France).

Similarly, ANOVA was conducted on the relative quantities of volatile compounds identified by GC-TOF-MS representing the three treatments, i.e., M3 control (not stored) and M3 stored at 40 °C in the vials and sachets. PCA, based on the correlation matrix, was also conducted using XLStat software.

## 4. Conclusions

The shelf-life stability of pre-formulated, single-serve iced tea powder products is crucial, as this will determine their physical properties, retention of the bioactive/volatile compounds, and sensory profiles. Changes that occur during shelf-life affect the consistency of a product related to its quality. The addition of xylitol instead of sugar makes the product suitable for diabetics, however, xylitol significantly increased the rate of dihydrochalcone degradation, indicating that a compromise would have to be made between stability and energy value, or an alternative low kJ sweetener should be investigated. The addition of citric acid monohydrate and ascorbic acid also resulted in overall decreased stability of the powder, especially when stored at high temperatures in semi-permeable sachets. From a sensory perspective, the chemical changes introduced in the samples stored in the sachets at 40 °C resulted in a sensory profile related to ‘fermented’ rooibos tea.

## Figures and Tables

**Table 1:** Moisture content, water activity (*a*_w_) and parameters for Guggenheim–Andersonde Boer (GAB) and Brunauer–Emmet–Teller (BET) models fitted to moisture adsorption data obtained at 25 °C for green rooibos extract powder and powder mixtures.

Powder	Interaction Average Heat Flow Error (µW/g) ^1^	Moisture Content (% Dry Basis)	*a*_w_	Parameters of GAB Model				Parameters of BET Model		
				*M*_0_^2^	*C*	*K*	R^2^	*M*_0_	*C*	R^2^
GRE ^3^	N/A ^4^	2.40 ± 0.14 b ^5^	0.176 ± 0.006 c	3.41	10.1	1.2	0.9503	3.73	10.4	0.9981
IN50 ^6^	0.76	2.98 ± 0.10 a	0.149 ± 0.004 d	3.83	6.9	1.2	0.8690	3.80	9.2	0.9986
M1 ^7^	4.90	0.661 ± 0.036 c	0.290 ± 0.013 b	0.178	11.1	1.1	0.7799	0.224	7.1	0.9743
M2 ^8^	1.29	0.348 ± 0.035 e	0.323 ± 0.011 a	0.148	12.5	1.4	0.7745	0.263	5.5	0.9733
M3 ^9^	4.85	0.489 ± 0.035 d	0.321 ± 0.010 a	0.154	11.7	1.4	0.7877	0.244	6.7	0.9955
M4 ^10^	3.13	0.517 ± 0.012 d	0.322 ± 0.022 a	0.184	11.1	1.1	0.7989	0.240	7.4	0.9721
M5 ^11^	3.45	0.596 ± 0.016 cd	0.333 ± 0.004 a	0.154	10.0	1.4	0.7287	0.255	5.7	0.9849

^1^ Difference between the heat flow of actual rooibos powder mixtures and the theoretical zero interaction curve of the ingredients. ^2^ Monolayer moisture content (% dry basis). ^3^ Green rooibos extract. ^4^ Measurement of interaction not possible with a single ingredient. ^5^ Means in the same column with the same letter are not significantly different (*p* ≥ 0.05). ^6^ GRE microencapsulated with inulin in 1:1 (m/m) ratio. ^7^ Mixture 1 (IN50 and sucrose). ^8^ Mixture 2 (IN50 and xylitol). ^9^ Mixture 3 (IN50, xylitol and citric acid). ^10^ Mixture 4 (IN50, xylitol and ascorbic acid). ^11^ Mixture 5 (IN50, xylitol, citric acid and ascorbic acid).

**Table 2:** Aspalathin degradation (%) and degradation rate constants (K) of the first order model ^1^ fitted to the experimental data representing aspalathin degradation in green rooibos extract powder and powder mixtures during storage at 30 °C/65% RH and 40 °C/65% RH for 12 months in glass vials and 5 months in semi-permeable sachets.

Powder	Sealed Glass Vials						Semi-Permeable Sachets			
	30 °C/65% RH			40 °C/65% RH			30 °C/65% RH		40 °C/65% RH	
	% Decrease (5 Months)	% Decrease (12 Months)	K (months^−1^)	% Decrease (5 Months)	% Decrease (12 Months)	K (months^−1^)	% Decrease (5 Months)	K (months^−1^)	% Decrease (5 Months)	K (months^−1^)
GRE ^2^	3.6 ± 0.5 c ^3^	6.7 ± 1.3 d	^4^	6.2 ± 0.5 d	17.8 ± 0.8 f	0.019 ± 0.003 e	4.9 ± 1.1 e	^4^	18.5 ± 0.2 f	0.035 ± 0.003 e
IN50 ^5^	1.9 ± 1.6 c	7.3 ± 0.3 d	^4^	3.7 ± 0.4 d	9.7 ± 0.4 g	^4^	0.3 ± 0.7 f	^4^	4.4 ± 0.7 g	^4^
M1 ^6^	15.3 ± 1.5 b	17.7 ± 1.6 c	0.016 ± 0.003 c	34.0 ± 2.0 c	50.1 ± 1.0 e	0.069 ± 0.005 d	25.2 ± 2.1 c	0.044 ± 0.008 b	39.8 ± 2.8 e	0.090 ± 0.018 d
M2 ^7^	12.1 ± 2.9 b	34.3 ± 3.0 b	0.040 ± 0.002 b	40.8 ± 2.3 b	76.9 ± 0.6 d	0.114 ± 0.007 c	21.0 ± 2.1 d	0.035 ± 0.005 b	61.5 ± 0.8 c	0.149 ± 0.014 b
M3 ^8^	11.9 ± 2.6 b	50.6 ± 2.3 a	0.056 ± 0.006 a	41.7 ± 1.5 b	87.0 ± 0.5 b	0.140 ± 0.003 b	29.9 ± 1.9 b	0.048 ± 0.012 b	50.2 ± 2.4 d	0.114 ± 0.006 c
M4 ^9^	27.4 ± 1.4 a	35.0 ± 2.7 b	0.040 ± 0.003 b	54.1 ± 1.3 a	85.5 ± 0.7 c	0.156 ± 0.0041a	41.2 ± 2.6 a	0.088 ± 0.011 a	82.1 ± 1.1 a	0.283 ± 0.010 a
M5 ^10^	25.9 ± 3.6 a	36.8 ± 3.0 b	0.040 ± 0.004 b	54.2 ± 5.3 a	90.3 ± 0.4 a	0.158 ± 0.012 a	41.9 ± 1.4 a	0.087 ± 0.011 a	67.0 ± 2.7 b	0.167 ± 0.003 b

^1^ *C* = *C*_0_ exp(-*Kt*), where *C* is aspalathin (g/100 g extract, d.b.), *C*_0_ is initial aspalathin (g/100 g extract, d.b.), *t* is the time in months and *K* is the reaction rate constant. ^2^ Green rooibos extract. ^3^ Means in the same column with the same letter are not significantly different (p ≥ 0.05). ^4^ Less than 10% change in aspalathin content observed-not suitable for kinetic modelling. ^5^ GRE microencapsulated with inulin in 1:1 (m/m) ratio. ^6^ Mixture 1 (IN50 and sucrose). ^7^ Mixture 2 (IN50 and xylitol). ^8^ Mixture 3 (IN50, xylitol, and citric acid). ^9^ Mixture 4 (IN50, xylitol, and ascorbic acid). ^10^ Mixture 5 (IN50, xylitol, citric acid, and ascorbic acid).

**Table 3:** Objective colour measurements (L*, a*, b*) and calculated C* ^1^, h ^2^ and ΔE ^3^ for M3 ^4^ reconstituted in water (63 g powder/L) before storage (control, ctrl) and after storage at 30 °C/65% RH and 40 °C/65% relative humidity for 1 month in glass sealed vials (v) and semi-permeable sachets (s).

Storage Condition	L*	a*	b*	C*	h	ΔE
ctrl	54.48 ± 0.79 b ^5^	4.81 ± 0.18 e	24.96 ± 0.28 e	25.15 ± 0.28 e	78.97 ± 0.40 a	0.00 ± 0.00 e
s_30 °C	55.83 ± 0.56 a	5.54 ± 0.31 c	27.65 ± 0.24 c	28.20 ± 0.27 c	78.68 ± 0.57 ab	3.39 ± 0.32 c
s_40 °C	51.01 ± 0.50 d	9.93 ± 0.15 a	36.04 ± 0.18 a	37.38 ± 0.20 a	74.60 ± 0.19 d	12.93 ± 0.27 a
v_30 °C	54.24 ± 0.54 b	5.22 ± 0.11 d	25.27 ± 0.35 d	25.80 ± 0.36 d	78.32 ± 0.11 b	0.88 ± 0.42 d
v_40 °C	53.53 ± 0.41 c	6.83 ± 0.25 b	28.33 ± 0.50 b	29.04 ± 0.51 b	77.33 ± 0.44 c	4.10 ± 0.56 b

^1^ Chroma. ^2^ Hue. ^3^ Colour difference. ^4^ Contains green rooibos extract microencapsulated with inulin in 1:1 ratio (m/m), xylitol, and citric acid. ^5^ Means in the same column with the same letter are not significantly different (*p* ≥ 0.05).

**Table 4:** Volatile compounds tentatively identified by comparison of calculated and literature retention indices (RI_cal_^1^ and RI_lit_, respectively) for M3 ^2^ reconstituted in water (63 g powder/L), before storage and after storage at 40 °C/65% relative humidity for 1 month in glass sealed vials and semi-permeable sachets.

Peak No.	Compound Name	RI_cal_^3^	RI_lit_^4^	Aroma Description ^5^
**1**	hexanal	1060	1083	green, grass, fatty
**2**	β-myrcene	1129	1161	spicy, balsamic, plastic
**3**	cumene	1137	1180	not available
**4**	limonene	1161	1213	citrus, camphor
**5**	2-hexenal	1196	1213	green, apple
**6**	2-pentylfuran	1202	1231	fruity, green, earthy
**7**	ethyl hexanoate	1208	1233	fruity, apple peel, pineapple
**8**	cymene	1239	1275	terpenic, fresh citrus, solvent,
**9**	octanal	1264	1289	aldehydic, fat, citrus, green
**10**	1-octen-3-one	1278	1300	earthy, mushroom, metal
**11**	(*Z*)-2-heptenal	1302	1322	green, fat, rancid
**12**	6-methyl-5-hepten-2-one	1316	1338	citrus, lemongrass, apple
**13**	(*E*)-2-octenal	1408	1429	fatty, fresh cucumber, green
**14**	1-octen-3-ol	1428	1450	earthy, soap, plastic
**15**	2-ethyl-1-hexanol	1467	1491	citrus, floral
**16**	(*E*)-2-nonenal	1515	1534	fatty, green cucumber
**17**	linalool	1526	1547	floral
**18**	1-octanol	1535	1557	waxy, moss, nut
**19**	(*E*,*Z*)-2,6-nonadienal	1566	1584	green, cucumber, leaf green, fat
**20**	6-methyl-3,5-heptadien-2-one	1577	1602	spicy, cinnamon, woody
**21**	2,5,5,8α-tetramethyl-3,5,6,8α-tetrahydro-2H-chromene	1585	1611	not available
**22**	(*E*)-2-decenal	1623	1644	fatty, waxy, coriander, green
**23**	1-nonanol	1638	1660	floral, fatty, dusty, oily
**24**	2-undecenal	1732	1751	fruity-sweet, orange peel
**25**	(*E*)-β-damascenone	1800	1823	fruity, apple, honey, tobacco
**26**	hexanoic acid	1824	1846	fatty, sour, sweat
**27**	geranyl acetone	1835	1859	floral, citrus, magnolia
**28**	α-calacorene	1892	1919	woody
**29**	3-(2,6,6-trimethyl-1-cyclohexen-1-yl-2-propenal)	1902	1936	not available
**30**	heptanoic acid	1931	1950	cheesy, rancid sour
**31**	octanoic acid	2037	2060	fatty, rancid, oily, vegetable
**32**	nonanoic acid	2143	2171	waxy, green, fatty
**33**	cadalene	2200	2233	not found
**34**	*n*-decanoic acid	2250	2276	fatty, rancid
**35**	3,5-di-tert-butylphenol	2291	2319	not found
**36**	dodecanoic acid	2462	2498	fatty, coconut, metal
**37**	hexadecanoic acid	2884	2915	waxy, creamy

^1^ RI_cal_ reported as average of all samples. ^2^ Contains green rooibos extract microencapsulated with inulin in 1:1 ratio (m/m), xylitol, and citric acid. ^3^ Experimental retention index (calculated by processing software (ChromaTOF^®^-GC-TOF-MS)). ^4^ Retention index from literature (NIST 11 MS library). ^5^ Aroma descriptors obtained from databases that supply information to the flavour industry (http://www.thegoodscentscompany.com and https://www.flavornet.org/flavornet.html, accessed on 6 July 2021.

**Table 5:** Components (%) of green rooibos extract powder and powder mixtures containing GRE ^1^ or IN50 ^2^ as the nutraceutical ingredient and various food grade ingredients including sugar or xylitol and acids.

Ingredient	Rooibos Powders						
	GRE	IN50	M1	M2	M3	M4	M5
GRE	100.00	0.00	0.00	0.00	0.00	0.00	0.00
IN50	0	100.00	5.54	5.54	5.54	5.54	5.54
Sucrose	0.00	0.00	94.46	0.00	0.00	0.00	0.00
Xylitol	0.00	0.00	0.00	94.46	92.58	94.15	92.26
Ascorbic acid	0.00	0.00	0.00	0.00	0.00	0.31	0.31
Citric acid	0.00	0.00	0.00	0.00	1.89	0.00	1.89

^1^ Green rooibos extract. ^2^ GRE microencapsulated with inulin in 1:1 (m/m) ratio.

## Data Availability

The data presented in this study are available upon request from the corresponding author.

## Supplementary Materials

The following are available online. Table S1: Nothofagin degradation (%) and average degradation rate constants (K) of the first order model fitted to the experimental data representing nothofagin degradation in green rooibos extract powder and powder mixtures during storage at 30 °C,/65% RH and 40 °C/65% RH for 12 months in glass vials and 5 months in semi-permeable sachets, Table S2: Sensory attributes used during descriptive analysis of reconstituted M3 before and after storage for 1 month at 30 °C/65% RH and 40 °C/65% RH in sealed glass vials and semi-permeable sachets, Figure S1: X-ray powder diffractograms of green rooibos extract powder and powder mixtures (**a**) green rooibos extract (GRE), (**b**) GRE microencapsulated with inulin in 1:1 ratio (m/m) (IN50) and powder mixtures, (**c**) M1 (IN50 and sucrose), (**d**) M2 (IN50 and xylitol), (**e**) M3 (IN50, xylitol and citric acid), (**f**) M4 (IN50, xylitol and ascorbic acid) and (**g**) M5 (IN50, xylitol, ascorbic and citric acid), before storage and after storage at 40 °C/65% RH for 12 months in glass vials and 5 months in semi-permeable sachets, Figure S2: Moisture content (**a**) and water activity (**b**) of green rooibos extract (GRE), GRE microencapsulated with inulin in 1:1 ratio (m/m) (IN50) and powder mixtures M1 (IN50 and sucrose), M2 (IN50 and xylitol), M3 (IN50, xylitol and citric acid), M4 (IN50, xylitol and ascorbic acid) and M5 (IN50, xylitol, ascorbic acid and citric acid) throughout storage at (**1**) 30 °C/65% RH and (**2**) 40 °C/65% RH for 12 months in sealed glass vials, Figure S3: Differential scanning calorimetry thermograms of green rooibos extract powder and powder mixtures (**a**) green rooibos extract (GRE), (**b**) GRE microencapsulated with inulin in 1:1 ratio (m/m) (IN50) and powder mixtures (**c**) M1 (IN50 and sucrose), (**d**) M2 (IN50 and xylitol), (**e**) M3 (IN50, xylitol and citric acid), (**f**) M4 (IN50, xylitol and ascorbic acid) and (**g**) M5 (IN50, xylitol, ascorbic and citric acid), before storage and after storage at 40 °C/65% RH for 12 months in glass vials and 5 months in semi-permeable sachets.
