# Supplementary material for: Shelf-Life Stability of Ready-to-Use Green Rooibos Iced Tea Powder—Assessment of Physical, Chemical, and Sensory Properties
Source: Molecules. 2021 Aug 30;26(17):5260. doi: 10.3390/molecules26175260 (PMC8433966; doi:10.3390/molecules26175260)
Supplement: Supplementary file 1 [file molecules-26-05260-s001.zip › molecules-1338504-supplementary.pdf]

SUPPLEMENTARY MATERIAL

Shelf-life Stability of Ready-To-Use Green Rooibos Iced Tea Powder – Assessment of Physical, Chemical and Sensory Properties

Chantelle Human [1,*], Dalene de Beer [1,2], Magdalena Muller [2], Marieta van Der Rijst [3], Marique Aucamp [4], Andreas Tredoux [5], André de Villiers [5], Elizabeth Joubert [1,2,]

[1] Plant Bioactives Group, Post-Harvest and Agro-Processing Technologies, Agricultural Research Council (Infruitec-Nietvoorbij), Private Bag X5026, Stellenbosch 7599, South Africa; HumanC@arc.agric.za (C.H.); DBeerD@arc.agric.za (D.d.B.); JoubertL@arc.agric.za (E.J.)

[2] Department of Food Science, Stellenbosch University, Private Bag X1, Matieland (Stellenbosch) 7600, South Africa; mm7@sun.ac.za (M.M.)

[3] Biometry Unit, Agricultural Research Council, Private Bag X5026, Stellenbosch 7599, South Africa; VanDerRijstM@arc.agric.za (M.v.D.R.)

[4] School of Pharmacy, University of the Western Cape, Private Bag X17, Bellville 7535, South Africa; maucamp@uwc.ac.za (M.A.)

[5] Department of Chemistry and Polymer Science, Stellenbosch University, Private Bag X1, Matieland (Stellenbosch) 7600, South Africa; atredoux@sun.ac.za (A.T.); ajdevill@sun.ac.za (A.d.V)

* Correspondence: HumanC@arc.agric.za; Tel.: (021)-809-3416

Table S1. Nothofagin degradation (%) and average degradation rate constants (K) of the first order model[1] fitted to the experimental data representing nothofagin degradation in green rooibos extract powder and powder mixtures during storage at 30 °C/65 % RH and 40 °C/65 % RH for 12 months in glass vials and 5 months in semi-permeable sachets.

Powder	Sealed glass vials						Semi-permeable sachets					
	30°C/65% RH			40°C/65% RH			30°C/65% RH			40°C/65% RH		
	% Decrease (12 months)	K (month^{-1})	R^2_{adj}[2]	% Decrease (12 months)	K (month^{-1})	R^2_{adj}	% Decrease (5 months)	K (month^{-1})	R^2_{adj}	% Decrease (5 months)	K (month^{-1})	R^2_{adj}
GRE[3]	8.3±0.6 d[4]	[5]	[5]	20.2±1.9 d	0.020±0.002 e	0.9164	5.3±1.4 e	[5]	[5]	17.3±2.2 f	0.035±0.003 f	0.8434
IN50[6]	10.2±0.9 d	[5]	[5]	12.1±1.6 d	[5]	[5]	0.4±0.5 f	[5]	[5]	3.3±0.4 g	[5]	[5]
M1[7]	18.9±1.9 c	0.017±0.000 c	0.6901	48.9±1.2 c	0.069±0.002 d	0.9453	24.1±2.1 c	0.047±0.013 bc	0.8171	37.6±6.2 e	0.087±0.009 e	0.9026
M2[8]	36.0±3.3 b	0.038±0.002 b	0.8641	76.4±0.6 b	0.113±0.003 c	0.9816	19.9±3.3 d	0.038±0.006 c	0.6979	59.5±1.3 c	0.143±0.005 c	0.9484
M39	48.5±2.9 a	0.058±0.001 a	0.9095	88.1±0.6 b	0.145±0.002 b	0.9357	29.1±3.0 b	0.059±0.005 b	0.7822	47.7±3.3 d	0.114±0.005 d	0.8308
M4[10]	35.7±3.5 b	0.041±0.005 b	0.7896	85.3±0.5 a	0.153±0.003 a	0.9786	39.7±4.3 a	0.082±0.009 a	0.9327	80.2±2.2 a	0.269±0.008 a	0.9560
M5[11]	33.7±3.3 b	0.035±0.003 b	0.8754	90.7±0.7 a	0.156±0.002 a	0.9802	40.8±2.1 a	0.083±0.007 a	0.8619	66.0±1.9 b	0.174±0.007 b	0.9300

[1] First order model: $C = C_0\exp(-Kt)$, where C is aspalathin (g/100 g extract, d.b.), C_0 is initial aspalathin (g/100 g extract, d.b.), t is the time in months and K is the reaction rate constant.
[2] Adjusted correlation of coefficient.
[3] Green rooibos extract.
[4] Means in the same column with the same letter are not significantly different ($p \geq 0.05$).
[5] Less than 10% change in aspalathin content observed – not suitable for kinetic modelling.
[6] GRE microencapsulated with inulin in 1:1 (m/m) ratio.
[7] Mixture 1 (IN50 and sucrose).
[8] Mixture 2 (IN50 and xylitol).
[9] Mixture 3 (IN50, xylitol and citric acid).
[10] Mixture 4 (IN50, xylitol and ascorbic acid).
[11] Mixture 5 (IN50, xylitol, citric acid and ascorbic acid).

Table S2. Sensory attributes used during descriptive analysis of reconstituted M3[1] before and after storage for 1 month at 30°C/65% RH and 40°C/65% RH in sealed glass vials and semi-permeable sachets.

Attributes	Descriptions
Rooibos-woody aroma	Aromatics associated with dry bushes, stems and twigs of fermented rooibos herbal tea
Apple aroma	Sweet aromatics associated with apple pie or cooked apples
Fruity-sweet aroma	Aromatics associated with sweet/sour smell of non-specific fruit
Honey aroma	Aromatics associated with fragrance of fynbos honey or *Alyssum* flowers
Hay/dried grass aroma	Slightly sweet aroma associated with dried grass or hay
Grainy aroma	Aroma of grains, porridge or dog pellets
Seaweed aroma	Aromatics associated with seaweed lying in the sun, fishy aroma/flavour of omega-3 oil
Rubber/putty-like aroma	Aromatics associated with Band-Aid®, rubber or putty
Dusty aroma	Earthy aromatics associated with dry dirt roads
Sweet taste	Fundamental taste of sucrose
Sour taste	Fundamental taste of citric acid
Bitter taste	Fundamental taste of caffeine
Astringent mouthfeel	Dry puckering feeling/sensation on the tongue and other mouth surfaces
Synthetic sweet aftertaste	A lingering, synthetic sweet taste

[1] Contains green rooibos extract microencapsulated with inulin in a 1:1 ratio (m/m), xylitol and citric acid.

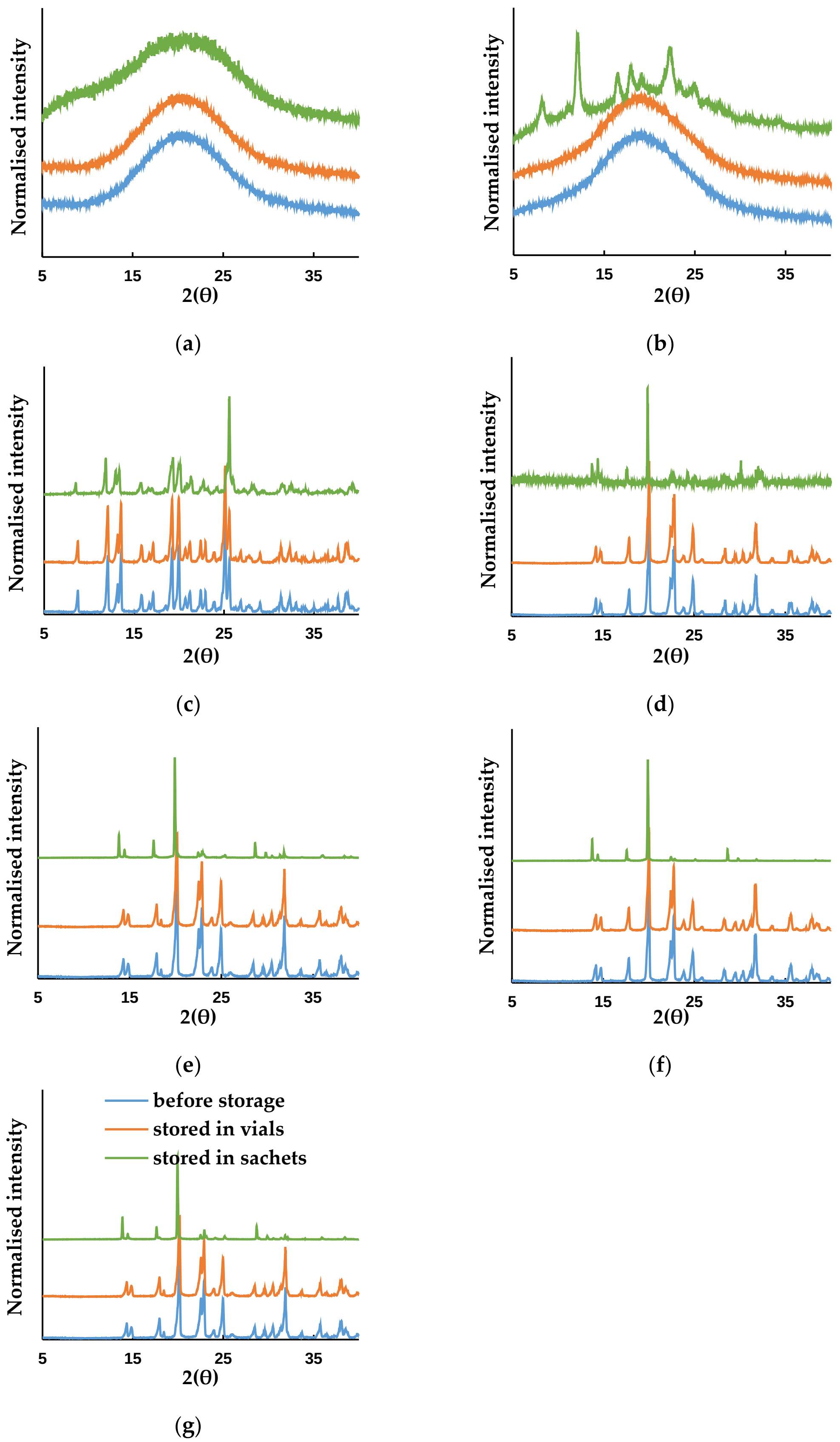

Figure S1. X-ray powder diffractograms of green rooibos extract powder and powder mixtures: (**a**) green rooibos extract (GRE), (**b**) GRE microencapsulated with inulin in 1:1 ratio (m/m) (IN50), (**c**) M1 (IN50 and sucrose), (**d**) M2 (IN50 and xylitol), (**e**) M3 (IN50, xylitol and citric acid), (**f**) M4 (IN50, xylitol and ascorbic acid) and (**g**) M5 (IN50, xylitol, ascorbic and citric acid), before storage and after storage at 40 °C/65% RH for 12 months in glass vials and 5 months in semi-permeable sachets.

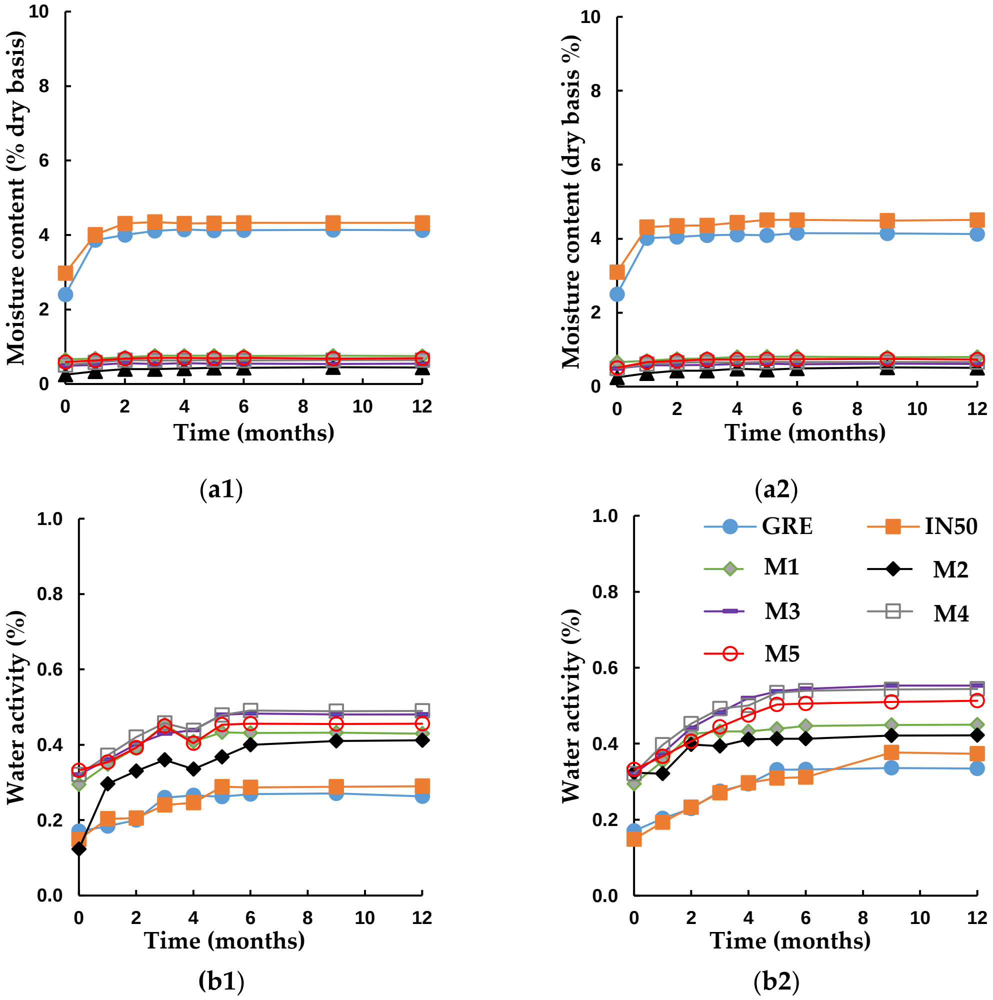

Figure S2. Moisture content (**a**) and water activity (**b**) of green rooibos powders: green rooibos extract (GRE), GRE microencapsulated with inulin in 1:1 ratio (m/m) (IN50), M1 (IN50 and sucrose), M2 (IN50 and xylitol), M3 (IN50, xylitol and citric acid), M4 (IN50, xylitol and ascorbic acid) and M5 (IN50, xylitol, ascorbic acid and citric acid) throughout storage at (**1**) 30°C/65% RH and (**2**) 40°C/65% RH for 12 months in sealed glass vials.

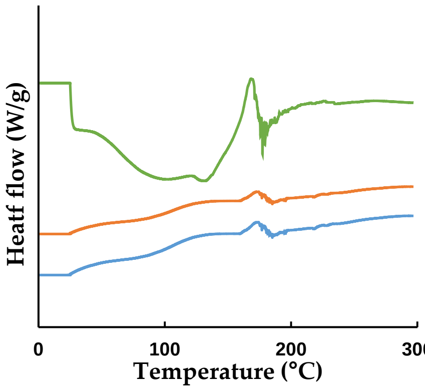

(**a**)

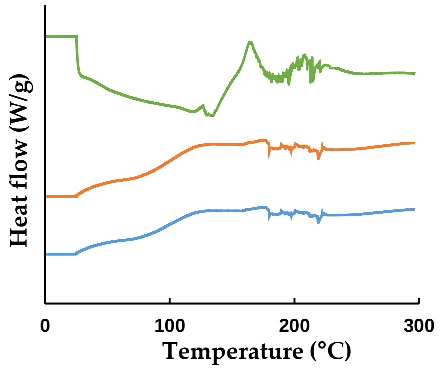

(**b**)

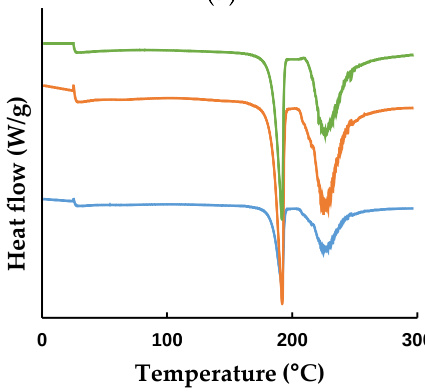

(**c**)

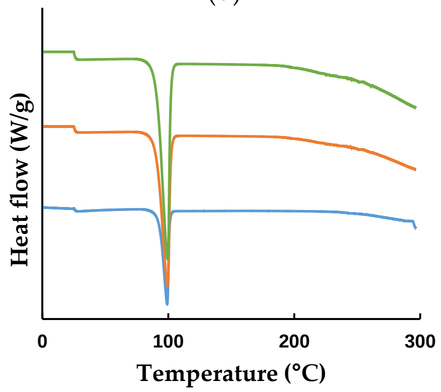

(**d**)

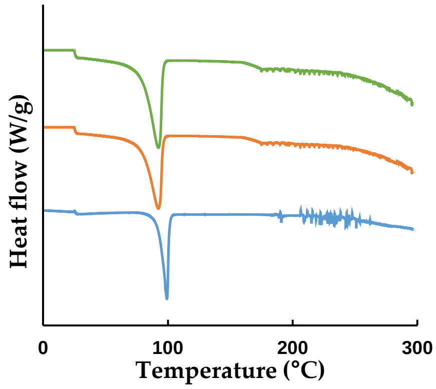

(**e**)

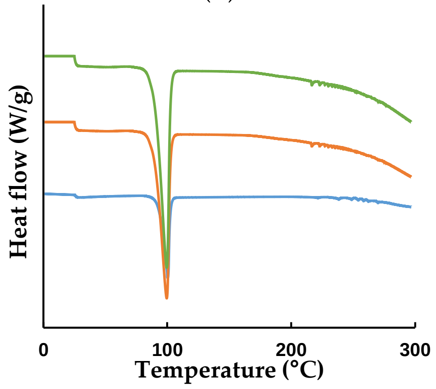

(**f**)

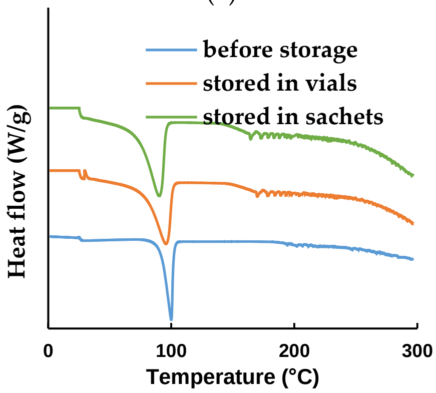

(**g**)

Figure S3. Differential scanning calorimetry thermograms of green rooibos extract powder and powder mixtures: (**a**) green rooibos extract (GRE), (**b**) GRE microencapsulated with inulin in 1:1 ratio (m/m) (IN50), (**c**) M1 (IN50 and sucrose), (**d**) M2 (IN50 and xylitol), (**e**) M3 (IN50, xylitol and citric acid), (**f**) M4 (IN50, xylitol and ascorbic acid) and (**g**) M5 (IN50, xylitol, ascorbic and citric acid), before storage and after storage at 40°C/65% RH for 12 months in glass vials and 5 months in semi-permeable sachets.
